# MaMPK3‐2 Phosphorylating and Stabilizing Transcriptional Repressor MaAS1 by Counteracting MaRNF4‐Mediated Ubiquitination During Banana Fruit Ripening

**DOI:** 10.1111/pbi.70769

**Published:** 2026-10-13

**Authors:** Ting‐Ting Luo, Ying‐Ying Yang, Hai‐Ke Tan, Wen Song, Wei Wei, Wei Shan, Jian‐Fei Kuang, Jian‐Ye Chen, Wang‐Jin Lu

**Affiliations:** ^1^ College of Horticulture South China Agricultural University Guangzhou China; ^2^ Department of Horticulture, College of Agronomy Jiangxi Agricultural University Nanchang China

**Keywords:** banana, fruit ripening, phosphorylation, PTM interaction, ubiquitination

## Abstract

The ripening of fleshy fruits is strongly regulated by a complex interplay between transcriptional regulation and post‐translational modifications (PTMs). However, the specific PTM regulatory networks involved in fruit ripening remain poorly understood. In this study, we identify MaAS1, an R2R3‐type MYB transcription factor, as a key negative regulator of banana fruit ripening. MaAS1 directly binds to the promoters of the ripening‐associated genes *MaACS1*, *MaPME23* and *MaEXPA15* and represses their transcription. The mitogen‐activated protein kinase MaMPK3‐2 phosphorylates MaAS1 at three specific sites (Ser61, Thr92 and Ser120), thereby enhancing its transcriptional repressor activity and protein stability and ultimately suppressing fruit ripening. In contrast, the ethylene‐induced E3 ubiquitin ligase MaRNF4 mediates the ubiquitination and degradation of MaAS1, thereby promoting ripening. Notably, phosphorylation by MaMPK3‐2 antagonises MaRNF4‐mediated ubiquitination, maintaining MaAS1 levels and regulating ripening progression. Collectively, these findings reveal a regulatory module involving MaMPK3‐2, MaAS1 and MaRNF4, ultimately highlighting the intricate interplay between phosphorylation and ubiquitination in controlling fruit ripening, offering critical insights into the integrated regulatory network interconnecting transcription and PTMs during fruit ripening.

## 1 Introduction

Banana fruit ripening is characterized by fruit softening and increased ethylene production, both of which are key physiological events (Kuang et al. 2021; Wei, Yang, et al. 2023). Pectin methylesterase (PME) and expansin (EXP) play crucial roles in cell wall disassembly: PME initiates pectin degradation by removing methyl groups (Xie et al. 2024), whereas EXP facilitates the slippage of cell wall fibres (Cosgrove 2015). Recent works have shown that the expression of *PME* and *EXP* genes is regulated by multiple transcription factor families, including NACs, ERFs and MYBs, which integrate developmental and hormonal signals to modulate cell wall remodelling during ripening (Cao et al. 2024). In parallel, ethylene biosynthesis is regulated by the rate‐limiting enzymes 1‐aminocyclopropane‐1‐carboxylic acid synthase (ACS) and oxidase (ACO) (Liu et al. 2020; Wu et al. 2022). Although the transcriptional regulation of *ACS* and *ACO* genes has been extensively studied, emerging evidence suggests that the transcription factors governing their expression, such as MYBs and ERFs, are regulated by post‐translational modifications, including phosphorylation and ubiquitination. These modifications fine‐tune ethylene output during ripening (Hou et al. 2017).

MYB transcription factors (TFs) constitute a large TF family in plants and are involved in diverse biological processes, including cell wall remodelling, secondary metabolism and hormone signalling. For example, CpMYB1 and CpMYB2 have been reported to inhibit papaya fruit softening (Fu et al. 2020), whereas MYB77 is involved in auxin and ABA signalling in citrus (Zhang et al. 2024). Recent studies have identified several R2R3‐MYB members in banana that modulate ripening through distinct mechanisms. For example, MaMYB69 acts as a transcriptional activator that is upregulated by ethylene and directly activates cell wall degradation genes, such as *MaPE*, *MaPL1*, *MaGAL* and *MaPG3*, thereby promoting fruit ripening (Liu et al. 2026). Additionally, MaMYB44 and MaMYB73 have been shown to interact and synergistically suppress cell wall metabolism and starch degradation genes, further illustrating the complexity of MYB‐mediated regulatory networks in banana (Luo et al. 2025, 2026). In apple, MdMYB44 positively regulates fruit crispness by directly activating the expression of the pectin methylesterase *MdMPE3* (Yang et al. 2025). In tomato, SlMYB73 negatively regulates ripening by recruiting the histone deacetylase HDA3, which suppresses the expression of ripening‐associated genes, including *ACO1*, *ACS2* and *PSY1* (Wang et al. 2026). However, the AS1 (MYB91) gene belongs to a distinct R2R3‐MYB subfamily, and its role in fruit ripening remains unexplored. It is essential for plant growth and development and has orthologs in maize (*ROUGH SHEATH2* [*RS2*]), *Arabidopsis* (*ASYMMETRIC LEAVES1* [*AS1*]) and *Antirrhinum* (*PHANTASTICA* [*PHAN*]), where these genes regulate organ development by repressing *KNOX* genes (Waites et al. 1998; Timmermans et al. 1999; Theodoris et al. 2003). Notably, these genes also exhibit functional divergence across species (Byrne et al. 2000; Guo et al. 2008). For example, OsMYB91 improves salt tolerance in rice (Zhu et al. 2015), and PagAS1a promotes xylem development in poplar (Li, Li, et al. 2025).

Phosphorylation and ubiquitination are key PTMs that regulate the stability and activity of TFs. The mitogen‐activated protein kinase (MAPK) cascade transmits signals through sequential phosphorylation events involving MAPKKK, MAPKK and MAPK (Wu et al. 2022; Zhang and Zhang 2022). Ubiquitination targets proteins for degradation based on the 26S proteasome, with E3 ubiquitin ligases providing substrate specificity (Akhter et al. 2023). RING‐type E3 ligases play essential roles in plant development (Shan et al. 2020; Wei, Yang, et al. 2023). The interplay between phosphorylation and ubiquitination represents a critical regulatory mechanism controlling protein stability and activity (Hu et al. 2017; Bai et al. 2024; Yu et al. 2024; Banerjee et al. 2026). In apple, phosphorylation promotes the ubiquitination and degradation of MdNAC72, thereby accelerating fruit softening (Wei, Liu, et al. 2023). In contrast, phosphorylation of banana MaMYB4 inhibits its ubiquitination, leading to ripening inhibition (Yang et al. 2023). Despite these findings, the precise molecular mechanisms underlying the regulation of fruit ripening via phosphorylation‐ubiquitination antagonism remain unclear.

In this study, we observed that MaAS1 functions as a transcriptional repressor that directly inhibits the expression of key genes involved in ethylene biosynthesis and cell wall modification in bananas. Furthermore, we found that a specific MAP kinase, MaMPK3‐2, phosphorylates MaAS1 at three sites, ultimately enhancing its repressor activity and stability. In contrast, the RING‐type E3 ligase, MaRNF4, targets MaAS1 for ubiquitination and subsequent degradation, thereby promoting the activation of ripening processes. Notably, phosphorylation by MaMPK3‐2 counteracts MaRNF4‐mediated ubiquitination, thereby maintaining MaAS1 levels and regulating ripening. This study provides insight into the intricate interplay between phosphorylation and ubiquitination in controlling fruit ripening.

## 2 Results

### 2.1 Identification and Characterization of MaAS1 From Banana Fruit

Kuang et al. (2021) reported that the number of downregulated TFs during ethylene‐induced banana fruit ripening was significantly higher than that of upregulated TFs. This finding indicated that transcriptional repressors might play a key role in regulating banana fruit ripening. Building on this finding, we focused on downregulated TFs and reanalyzed previously published transcriptome data to identify a strongly downregulated R2R3‐type MYB TF, designated MaAS1. Amino acid sequence alignment revealed that MaAS1 was highly similar to MYB91 orthologs from *Arabidopsis*, tomato and rice. MaAS1 consisted of the typical R2R3 domain and an EAR motif (Figure S1). To investigate the function of MaAS1, we treated unripe banana fruits with exogenous ethylene and compared them with untreated fruits. Ethylene treatment accelerated banana fruit ripening, leading to faster colour development and softening (Figure 1A,B) and an earlier climacteric ethylene peak (Figure 1C). *MaAS1* transcript levels remained high in naturally ripening fruits but decreased following ethylene treatment (Figure 1D). Functional assays in yeast and tobacco indicated that MaAS1 lacked transcriptional activation activity (Figure 1F,G). Subcellular localization assays confirmed that MaAS1 was localized in the nucleus (Figure 1E). These findings indicated that MaAS1 functioned as a transcriptional repressor and its expression was inversely correlated with banana fruit ripening.

**FIGURE 1:**
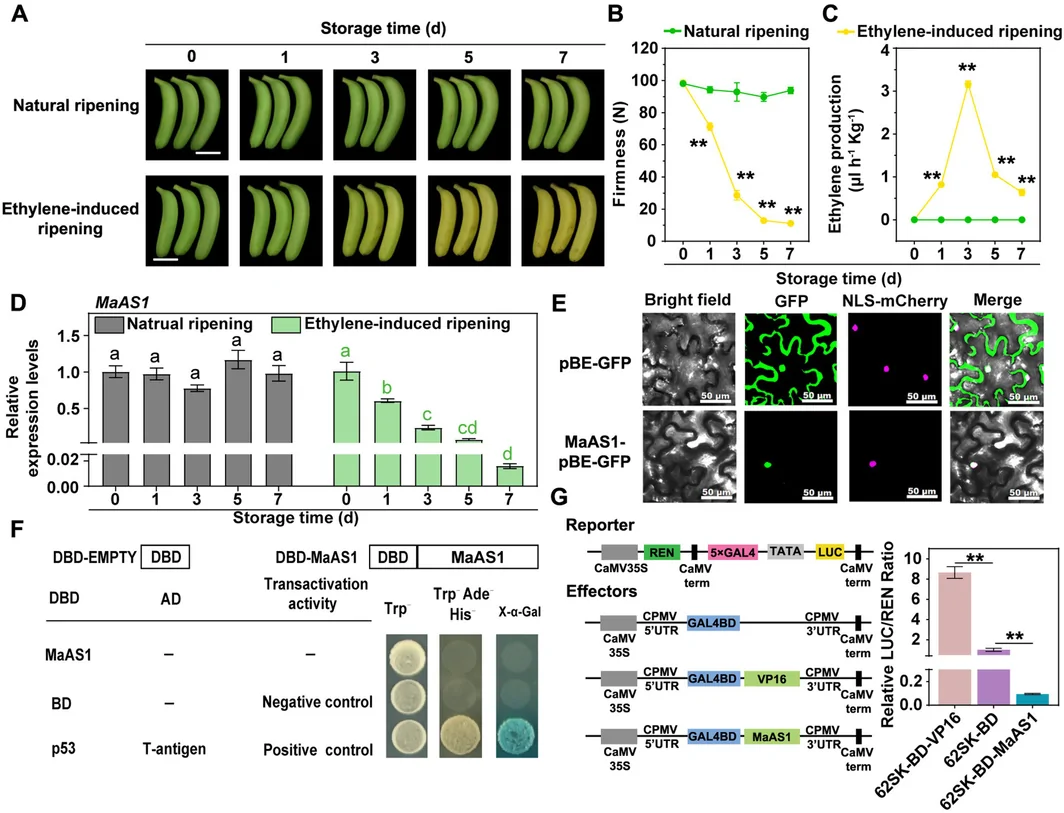
Phenotypic changes during banana fruit ripening and molecular characterization of MaAS1. (A) Phenotypes of banana fruits during natural and ethylene‐induced ripening. Scale bar = 5 cm. (B) Fruit firmness and (C) ethylene production during ripening. Data are presented as mean ± SE of three biological replicates. **Indicates a significant difference between natural and ethylene‐induced ripening (Student's *t*‐test, **p < 0.01). (D) Relative *MaAS1* transcript levels during natural and ethylene‐induced ripening. Values are presented as mean ± SE of three biological replicates. Different lowercase letters indicate significant differences among samples (one‐way ANOVA, *p* < 0.05). (E) Subcellular localization of MaAS1‐GFP in tobacco leaf cells. Subcellular localization was observed via fluorescence microscopy 36–48 h after infiltration. Nuclear localization signal (NLS)‐mCherry was used as a nuclear marker. Scale bar = 50 μm. (F) Transcriptional activation analysis of MaAS1 in yeast cells. The empty pGBKT7 vector was used as a negative control, whereas pGBKT7‐53 + pGADT7‐T served as a positive control. (G) Transcriptional repression activity of MaAS1 in tobacco leaves. The BD‐62SK vector served as a negative control, and BD‐62SK‐VP16 served as a positive control. Transcriptional activity was evaluated based on the relative LUC/REN ratio. Data are presented as mean ± SE of three biological replicates. **Indicates a significant difference (Student's *t*‐test, *p* < 0.01).

### 2.2 Genome‐Wide Identification of MaAS1 Target Genes

To identify potential binding sites and target genes of MaAS1, we performed DAP‐seq analysis using three biological replicates, obtaining 1493 overlapping peaks (Figure 2A). These peaks demonstrated high reproducibility (Figure S2), indicating a robust dataset. After distal intergenic peaks were filtered, 257 binding peaks were identified in promoter regions, representing 43.34% of the retained peaks (Figure 2B). MaAS1 binding sites were predominantly located within 1 kb upstream of the transcription start site (TSS) (Figure 2C), which in turn indicated a role in transcriptional regulation (Figure 2B). HOMER with standard settings identified an enriched motif: RMAACATBYT (Figure 2D). Fruit softening and ethylene production are characteristic ripening events of climacteric fruits. To delineate the regulatory role of MaAS1 in fruit ripening, we screened all banana gene promoters for the above motif and identified 2119 putative target genes (Data S2, Figure 2E). GO enrichment analysis indicated that these genes were mainly involved in biosynthesis, hormone signalling responses and regulation of plant growth and development (Figure 2F). We compared the 2119 putative target genes with genes upregulated in our fruit‐ripening transcriptome dataset (Data S1), identifying 827 overlapping genes (Figure 2G). Among these genes, six genes were associated with fruit ripening, including genes involved in cell wall modification and ethylene biosynthesis (Figure 2H). Considering potential functional redundancy, we selected three genes with the highest transcript abundance—*MaPME23*, *MaACS1* and *MaEXPA15*—as representative targets for further functional analysis.

**FIGURE 2:**
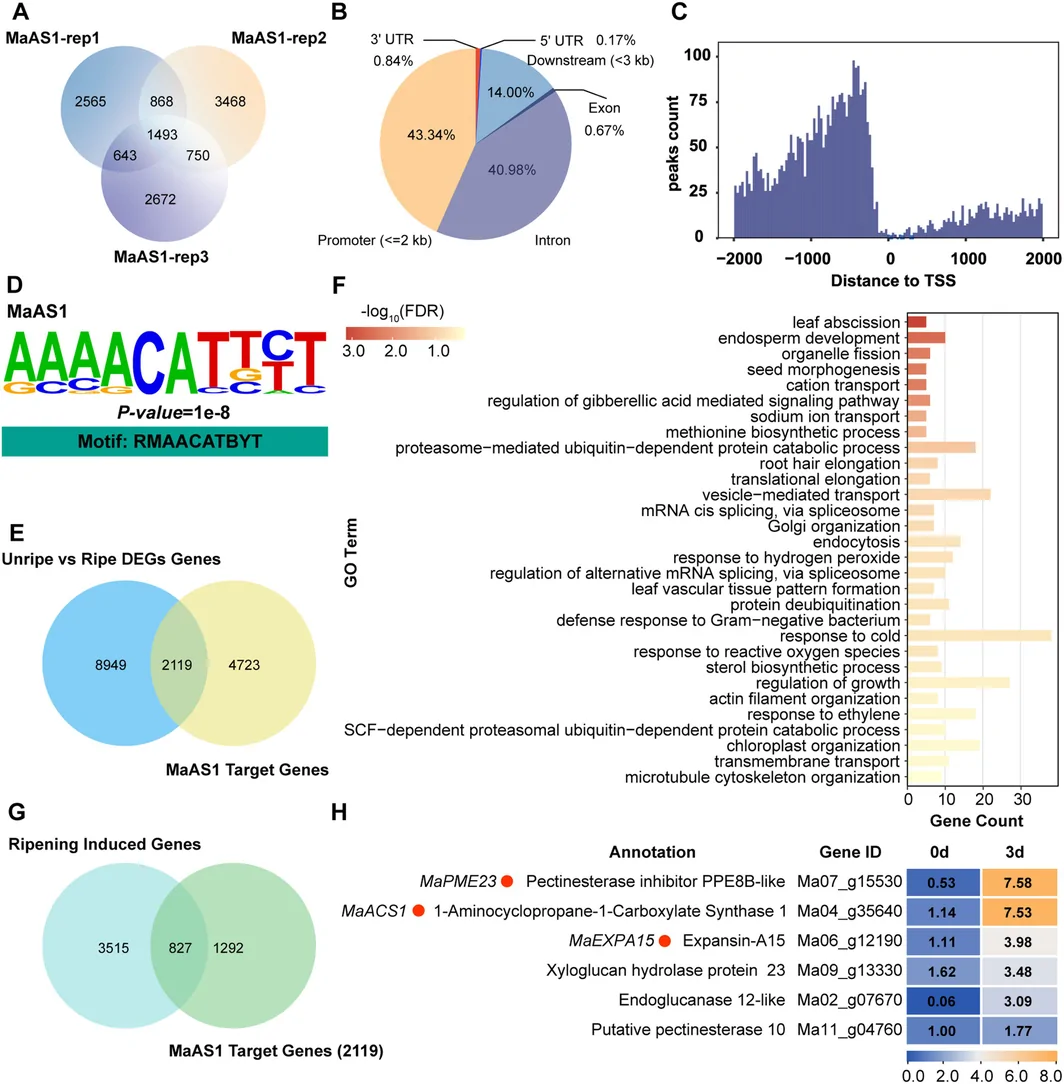
Genome‐wide identification of potential target genes of MaAS1. (A) 1493 high‐confidence MaAS1‐binding peaks, identified via DAP‐seq using three biological replicates. (B) Distribution of MaAS1‐binding sites across genic regions, including promoter (≤ 2 kb), 3′UTR, 5′UTR, downstream, exon and intron regions. (C) MaAS1‐binding sites, highly enriched in regions proximal to the TSS. (D) Enriched binding motif identified from MaAS1 target genes via DAP‐seq. (E) Venn diagram showing the overlap between MaAS1 target genes (identified via genome‐wide screening) and previously published RNA‐seq data on banana fruit ripening. (F) GO enrichment analysis of biological processes associated with MaAS1 target genes identified via genome‐wide screening. (G) Venn diagram showing the overlap between MaAS1 target genes and ripening‐induced DEGs identified from previously published RNA‐seq data on banana fruit ripening. (H) Heatmap showing the expression levels of fruit ripening‐related genes at 0 and 3 days after ethylene treatment. The heatmap was generated using log_2_(FPKM + 1) values.

### 2.3 MaAS1 Binding to the Promoters of *MaPME23* , *MaACS1* and *MaEXPA15* and Repressing Their Transcription

To further elucidate how MaAS1 regulates banana fruit ripening, we performed an electrophoretic mobility shift assay (EMSA) to confirm its direct binding to candidate target genes. Probes encompassing the specific motif (RMAACATBYT) were designed from the promoter regions of *MaPME23*, *MaACS1* and *MaEXPA15*. Recombinant GST‐MaAS1 protein exhibited specific binding to these probes in vitro, and the addition of competitive probes did not disrupt this interaction, whereas GST alone did not produce shifted bands (Figure 3A). To verify whether MaAS1 directly binds to the promoters of these target genes in vivo, we performed chromatin immunoprecipitation (ChIP)‐polymerase chain reaction (PCR) assays, using IgG as a negative control. As shown in Figure 3B, the promoter fragments of these target genes were significantly enriched in the DNA immunoprecipitated by the anti‐MaAS1 antibody compared to the IgG control (Figure 3B), demonstrating that MaAS1 can associate with the promoters of its target genes in vivo. Subsequently, we performed dual‐luciferase reporter assays (DLR) to investigate the effect of MaAS1 on the transcription of these genes. Expression of the MaAS1 effector significantly reduced LUC activity driven by the promoters of *MaPME23*, *MaACS1* and *MaEXPA15* (Figure 3C). These findings indicated that MaAS1 directly interacted with the promoter sequences of *MaPME23*, *MaACS1* and *MaEXPA15*, leading to repression of their transcription.

**FIGURE 3:**
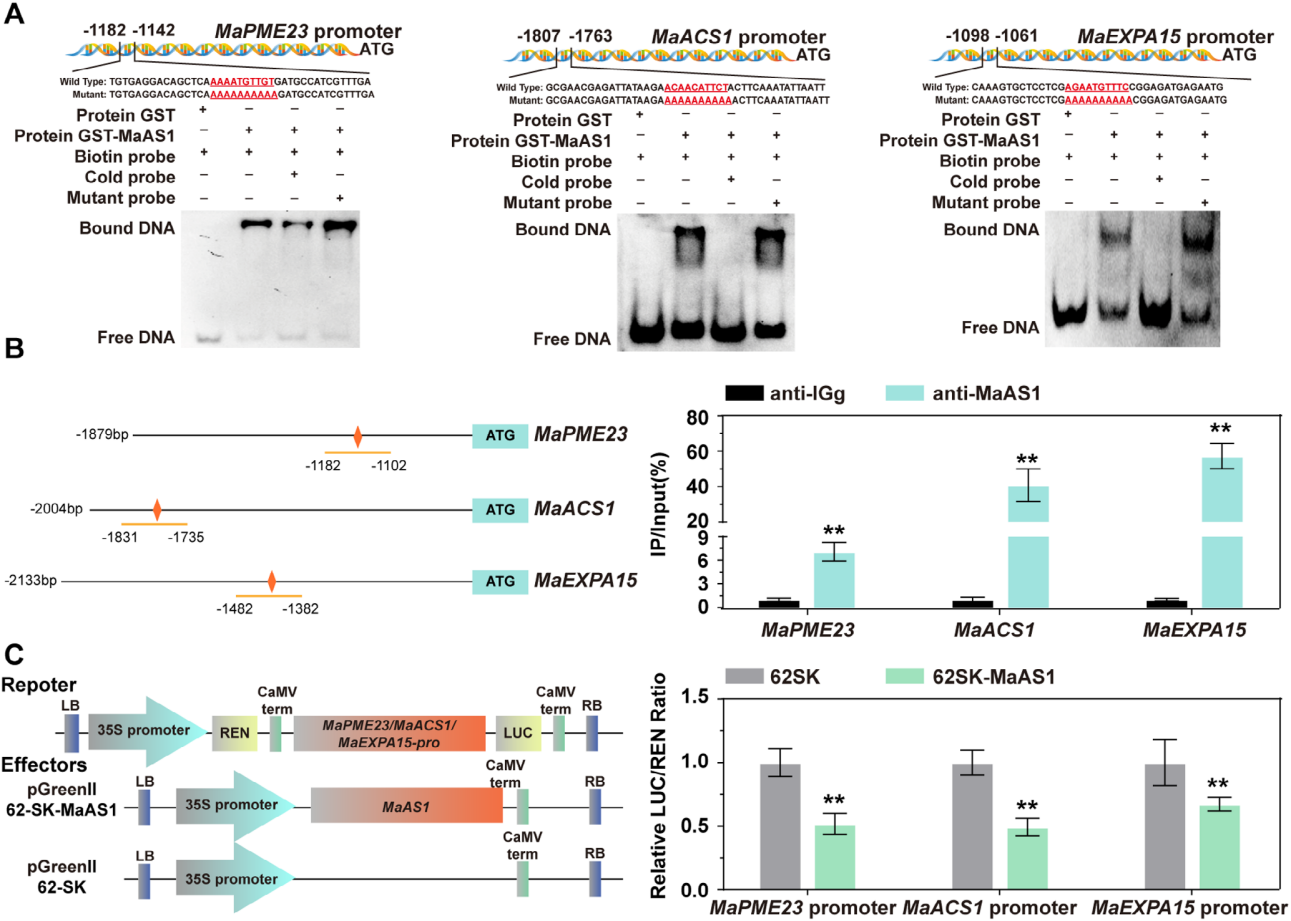
MaAS1 directly repressing banana fruit ripening by binding with *MaPME23*, *MaACS1* and *MaEXPA15*. (A) EMSA assay demonstrating the binding of MaAS1 to the promoters of *MaPME23*, *MaACS1* and *MaEXPA15*. DNA‐protein binding complexes were detected only in the absence of GST‐MaAS1 protein. ‘−’ and ‘+’ indicate the absence and presence of the corresponding component in the reaction system, respectively. (B) ChIP‐qPCR shows the in vivo binding of MaAS1 to the promoters of *MaPME23*, *MaACS1* and *MaEXPA15*. The data represent mean ± SE (*n* = 3 biological replicates). (C) DLR assay showing that MaAS1 represses the transcription of *MaPME23*, *MaACS1* and *MaEXPA15*. Transcriptional activity was determined based on the LUC/REN ratio. **Indicates a significant difference (Student's *t*‐test, **p < 0.01).

### 2.4 MaMPK3‐2 Phosphorylating MaAS1 to Enhance Its Repressor Activity and Stability

Accumulating evidence confirms that MPK‐mediated phosphorylation modulates MYB transcription factors across various plant species (Li et al. 2016; Lin et al. 2022; Yang et al. 2023). To investigate the regulation of MaAS1 stability via phosphorylation, we screened for interacting MPKs among 25 banana MPKs (Asif et al. 2014). Owing to the presence of multiple transcripts for several MPK genes, a total of 26 gene IDs were obtained (Yang et al. 2023). Integration with fruit‐ripening transcriptome data identified 13 candidate genes (Figure 4A), of which 6 were downregulated during banana fruit ripening, including *MaMPK9‐1*, *MaMPK11‐2A*, *MaMPK20‐4*, *MaMPK9‐4*, *MaMPK3‐2* and *MaMPK11‐3* (Figure 4B and Data S3). Bimolecular fluorescence complementation (BiFC) assays were performed to test pairwise interactions between MaAS1 and the six MPKs. YFP fluorescence, co‐localizing with the nuclear marker NLS‐mCherry, was detected exclusively when MaAS1 and MaMPK3‐2 were co‐expressed (Figure 4C). No YFP signal was observed in the other combinations (Figure S3). In pull‐down assays, MBP‐MaMPK3‐2 was specifically pulled down by GST‐MaAS1, but not by GST (Figure 4D). MaMPK3‐2 belongs to the group A MAPK family (Figure S4). Its transcript levels decreased during fruit ripening (Figure 4E), and its overexpression in tomato delayed ripening and repressed the expression of orthologs of MaAS1 target genes (Figure 5A–D), confirming its negative role in banana fruit ripening. To determine whether MaMPK3‐2 directly phosphorylates MaAS1, we conducted an in vitro phosphorylation assay. A mobility shift of phospho‐GST‐MaAS1 was detected via SDS‐PAGE in the presence of MBP‐MaMPK3‐2, and the shift was eliminated by treatment with alkaline phosphatase (AP) (Figure 5E). LC–MS/MS analysis subsequently identified three phosphorylation sites in MaAS1 (Ser61, Thr92 and Ser120) (Figure 5F).

**FIGURE 4:**
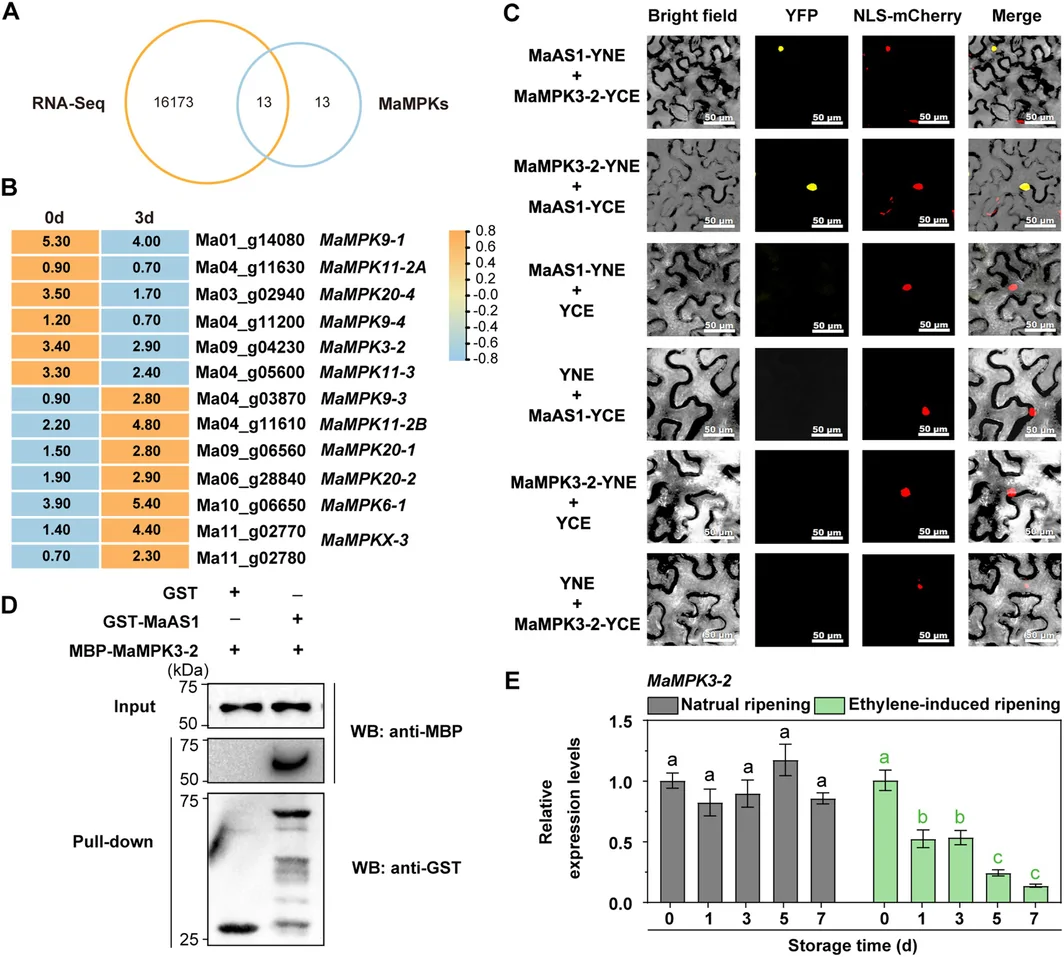
MaAS1 interacting with MaMPK3‐2 in vitro and in vivo. (A) Venn diagram showing the overlap between banana *MaMPKs* and RNA‐seq data. (B) Heatmap displaying the expression levels of the common *MaMPKs* at 0 and 3 days after ethylene treatment. Values are expressed as log_2_(FPKM + 1). (C) BiFC analysis confirming the interaction between MaAS1 and MaMPK3‐2. NLS‐mCherry was used as a nuclear marker. Scale bar = 50 μm. (D) In vitro pull‐down assay confirming the interaction between MaAS1 and MaMPK3‐2. Bound proteins were detected via Western blot using anti‐MBP and anti‐GST antibodies. ‘−’ and ‘+’ indicate the absence and presence of the corresponding component in the reaction system, respectively. (E) Relative expression levels of *MaMPK3‐2* during natural and ethylene‐induced ripening of banana fruit. Shown the value as the mean ± SE of three replicates. The letters indicate significant difference among different time point (one‐way ANOVA, *p* < 0.05).

**FIGURE 5:**
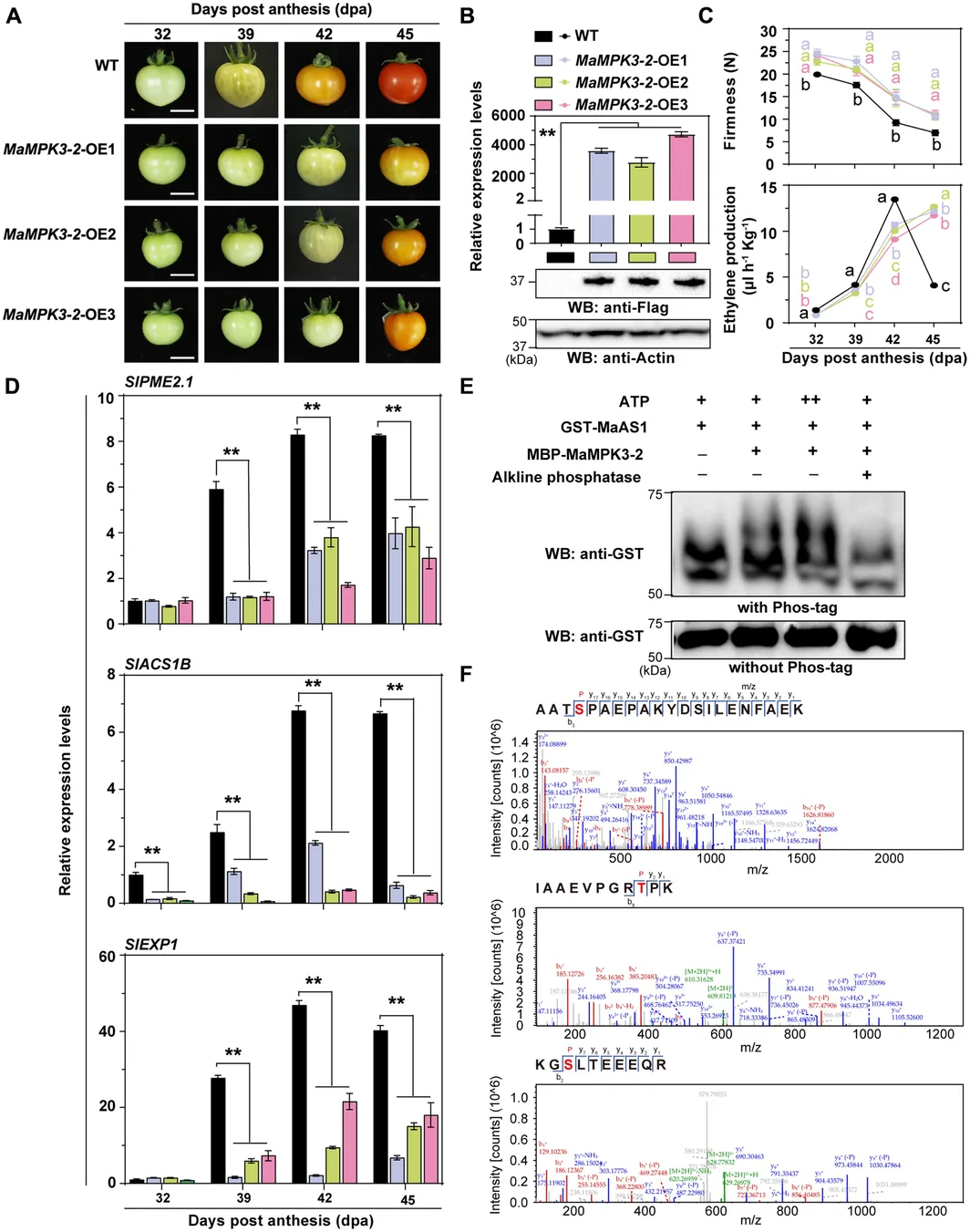
Functional validation of *MaMPK3‐2* and identification of phosphorylation sites of MaAS1. (A–C) Overexpression of *MaMPK3‐2* in tomato delays fruit ripening. (A) Fruit ripening process of wild‐type (WT) and three *MaMPK3‐2*‐OE lines. The images at the 32, 39, 42 and 45 days post‐anthesis (dpa) are shown. Bar, 1 cm. (B) Transgenic success was confirmed using RT‐qPCR and Western blot. (C) Tomato fruit firmness and ethylene production at different time points. The data were represented as mean ± SE of three replicates. The minuscules denote a significant different between WT and *MaMPK3‐2*‐OE lines (one‐way ANOVA, *p* < 0.05). (D) Relative mRNA abundance of *SlPME2.1*, *SlACS1B* and *SlEXP1* in WT and *MaMPK3‐2*‐OE lines. Present the value as mean ± SE of three replicates, the ** indicates a significant different (Student's *t*‐test, *p* < 0.01). (E) MaMPK3‐2 phosphorylating MaAS1 in vitro. Recombinant protein MBP‐MaMPK3‐2 was incubated with GST‐MaAS1 in a buffer containing ALP or ATP, resolved via Phos‐tag SDS‐PAGE, and then identified via Western blot using an anti‐GST antibody. ‘−’, ‘+’ and ‘++’ denote no component, standard dosage and double dosage, respectively. (F) Identification of three phosphorylation sites (Ser‐61, Thr‐92 and Ser‐120) in MaAS1 phosphorylated by MaMPK3‐2.

To evaluate the functional impact of these phosphorylation events, we created a phosphomimetic mutant (*MaAS1* ^*S61E, T92E, S120E*^) and a nonphosphorylatable mutant (*MaAS1* ^*S61A, T92A, S120A*^). Phosphomimetic mutants replace the phosphorylation sites with negatively charged amino acids such as glutamic acid (Glu) or aspartic acid (Asp) through site‐directed mutagenesis, whereas nonphosphorylatable mutants replace these residues with positively charged alanine (Ala) (Dóczi and Bögre 2018). Transient expression assays in tobacco revealed that the phosphomimetic mutant (*MaAS1* ^*S61E, T92E, S120E*^) exhibited significantly enhanced transcriptional repression activity compared with native MaAS1, whereas the nonphosphorylatable mutant (*MaAS1* ^*S61A, T92A, S120A*^) exhibited weakened repression (Figure 6A), indicating that phosphorylation enhanced the repressive function of MaAS1. Transient overexpression in banana fruit further confirmed that the phosphomimetic mutant (*MaAS1* ^*S61E, T92E, S120E*^) delayed degreening, softening and the ethylene peak more effectively compared with native MaAS1, whereas the nonphosphorylatable mutant (*MaAS1* ^*S61A, T92A, S120A*^) behaved similarly to the empty vector (Figure 6B–D). Similarly, in stable tomato transformants, the phosphomimetic mutant (*MaAS1* ^*S61E, T92E, S120E*^) further delayed fruit ripening, whereas the nonphosphorylatable mutant accelerated it (Figure 7A–C). Downstream target genes (*MaPME23*, *MaACS1* and *MaEXPA15*) were most strongly repressed by the phosphomimetic mutant and least repressed by the nonphosphorylatable mutant (Figure 6E), a pattern that was also conserved in tomato (Figure 7D).

**FIGURE 6:**
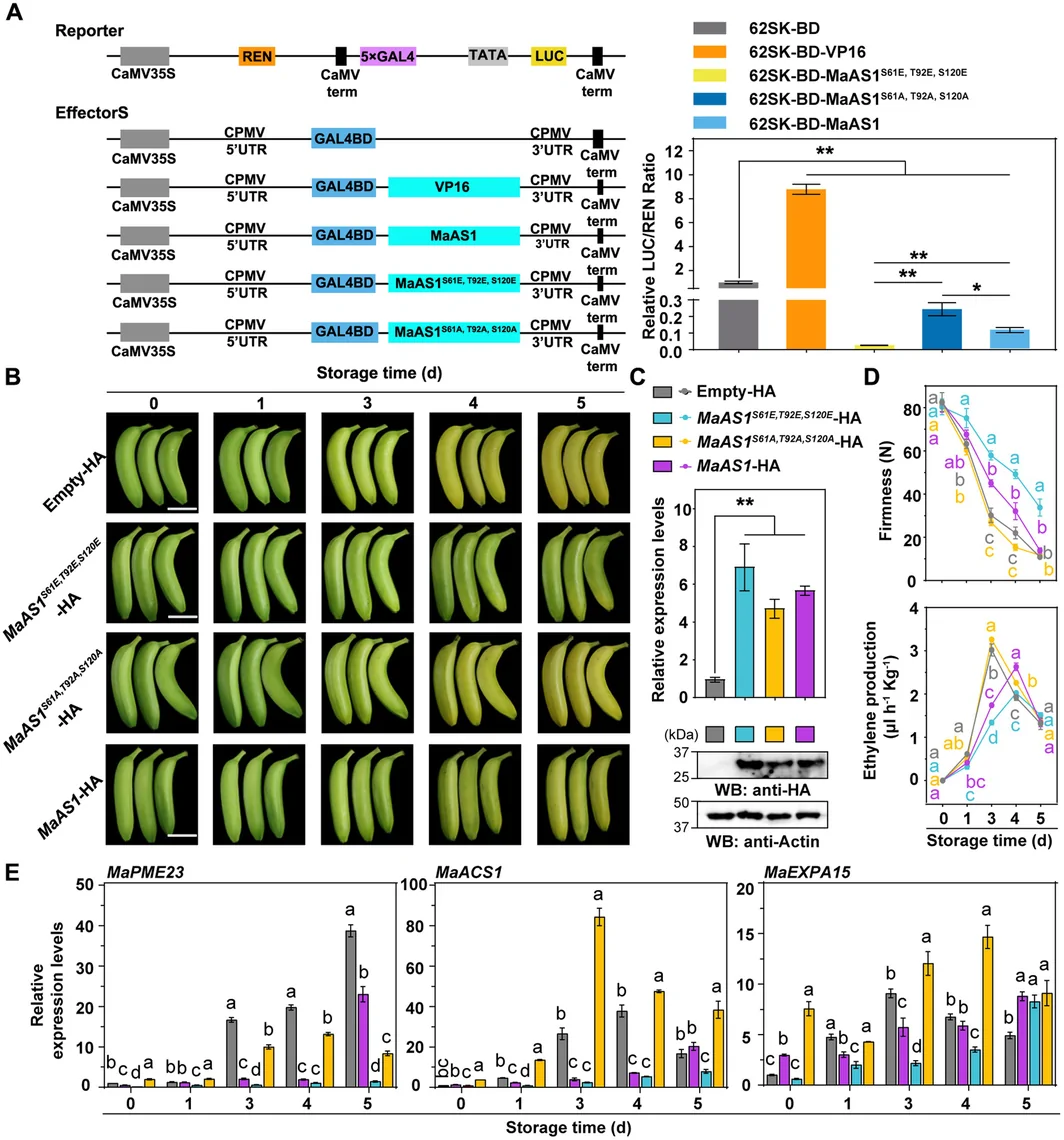
Phosphorylation of MaAS1 at Ser61, Thr92 and Ser120 repressing banana fruit ripening. (A) Transcriptional activity of *MaAS1*, *MaAS1* ^*S61E, T92E, S120E*^ and *MaAS1* ^*S61A, T92A, S120A*^ in *Nicotiana benthamiana* leaves. (B–D) Transient overexpression of empty vector, *MaAS1*, *MaAS1* ^*S61E, T92E, S120E*^ and *MaAS1* ^*S61A, T92A, S120A*^ in banana fruit. (B) Phenotypic differences among different treatments during ethylene‐induced ripening. Images were digitally extracted for comparison. Scale bar = 5 cm. (C) Overexpression of *MaAS1*, *MaAS1* ^*S61E, T92E, S120E*^ and *MaAS1* ^*S61A, T92A, S120A*^ in banana fruit, as detected via RT‐qPCR and Western blot. Data are presented as mean ± SE of three biological replicates. **Indicates a significant difference between treatments (Student's *t*‐test, *p* < 0.01). (D) Changes in fruit firmness and ethylene production during ripening. (E) Relative expression levels of *MaPME23*, *MaACS1* and *MaEXPA15* in *MaAS1*‐HA, *MaAS1* ^*S61E, T92E, S120E*^‐HA, *MaAS1* ^*S61A, T92A*, *S120A*^‐HA and empty vector banana fruit. Data are presented as mean ± SE of three biological replicates. Different letters indicate significant differences among treatments (one‐way ANOVA, *p* < 0.05).

**FIGURE 7:**
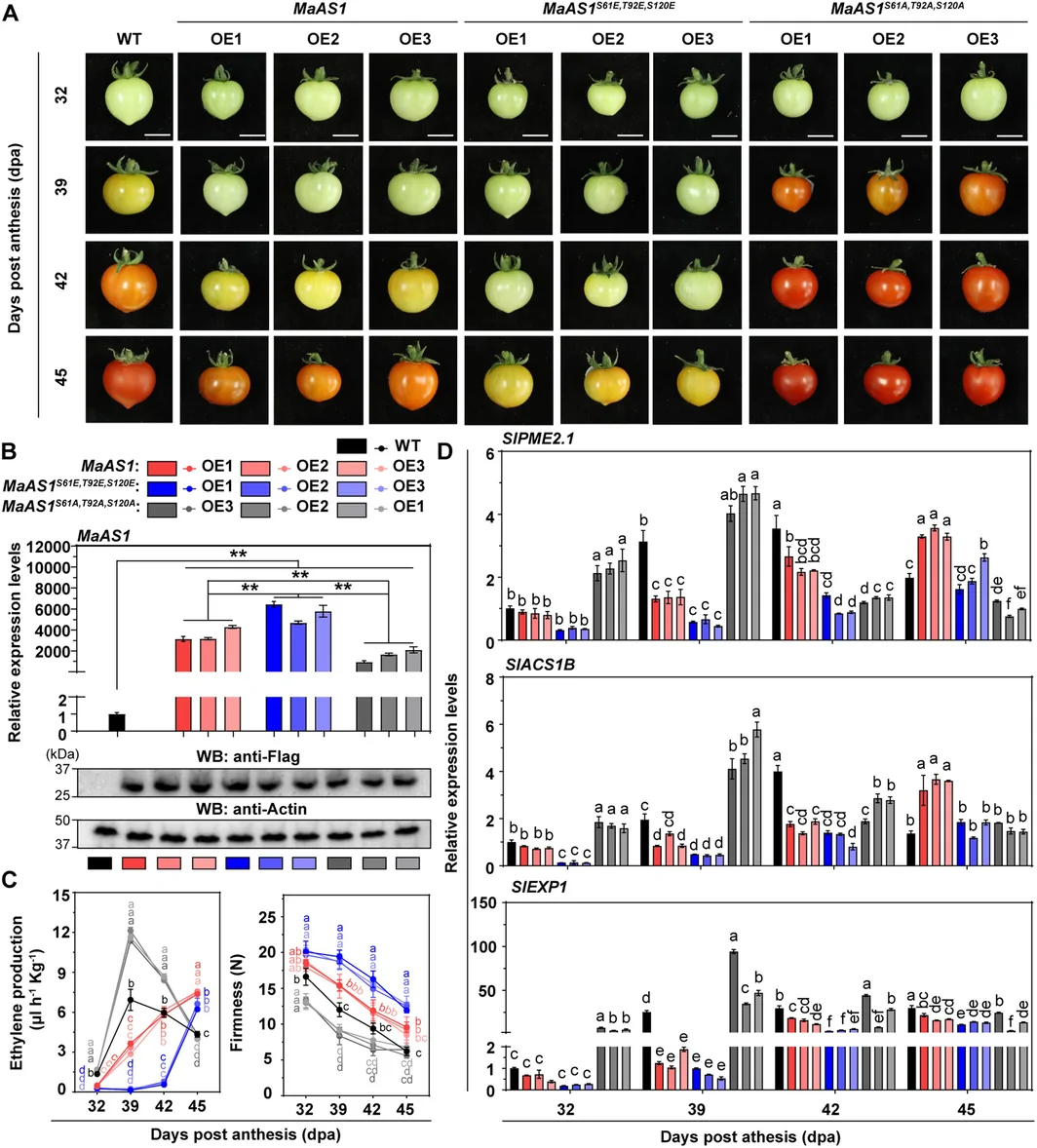
Functional characterization of *MaAS1* ^*S61E, T92E, S120E*^, *MaAS1* ^*S61A, T92A, S120A*^ and *MaAS1* in tomato fruit. (A) Tomato fruit images of M*aAS1*‐OE, *MaAS1* ^*S61E, T92E, S120E*^‐OE, *MaAS1* ^*S61A, T92A, S120A*^‐OE lines and WT at 32, 39, 42 and 45 dpa are shown. (B) Transgenic conformation was performed using RT‐qPCR and Western blot. Each value was expressed as mean ± SE of three replicates. The **indicates a significant difference (Student's *t*‐test, *p* < 0.01). (C) Changes of fruit firmness and ethylene production were observed in *MaAS1* ^*S61E, T92E, S120E*^‐OE lines, *MaAS1* ^*S61A, T92A, S120A*^‐OE, *MaAS1*‐OE lines and WT. (D) Relative expression levels of *SlPME2.1*, *SlACS1B* and *SlEXP1* in WT and *MaAS1*‐OE, *MaAS1* ^*S61E, T92E, S120E*^‐OE, *MaAS1* ^*S61A, T92A, S120A*^‐OE lines. The letters denote a significant difference (one‐way ANOVA, *p* < 0.05).

Notably, immunoblot analysis revealed that MaAS1 protein levels were significantly higher in the phosphomimetic mutant (*MaAS1* ^*S61E, T92E, S120E*^) than in the *MaAS1*‐HA fruit. In contrast, the nonphosphorylatable mutant (*MaAS1* ^*S61A, T92A, S120A*^) exhibited substantially reduced MaAS1 protein levels (Figure 6C). These results indicated that phosphorylation of MaAS1 at Ser61, Thr92 and Ser120 enhanced its transcriptional repression activity and protein stability, leading to repression of ripening‐related genes and delayed fruit ripening.

### 2.5 MaRNF4 Mediating Ubiquitination and Degradation of MaAS1 to Accelerate Fruit Ripening

The decreased MaAS1 levels observed in the nonphosphorylatable mutant prompted us to investigate whether MaAS1 was regulated by ubiquitin‐dependent degradation. Through Y2H screening, we identified the RING‐type E3 ubiquitin ligase MaRNF4 as an interacting protein (Figure 8A). This interaction was further confirmed via BiFC (Figure 8B) and GST pull‐down assays (Figure 8C), both in vivo and in vitro. The expression of *MaRNF4* gradually increased during fruit ripening (Figure S5A). In vitro ubiquitination assays revealed that MaRNF4 exhibited E3 ubiquitin ligase activity (Figure S5B) and directly ubiquitinated MaAS1 (Figure 8D). A cell‐free degradation assay (Figure 8E) and an in vivo ubiquitination assay (Figure 8F) further confirmed that MaRNF4 targets MaAS1 for degradation via the 26S proteasome pathway.

**FIGURE 8:**
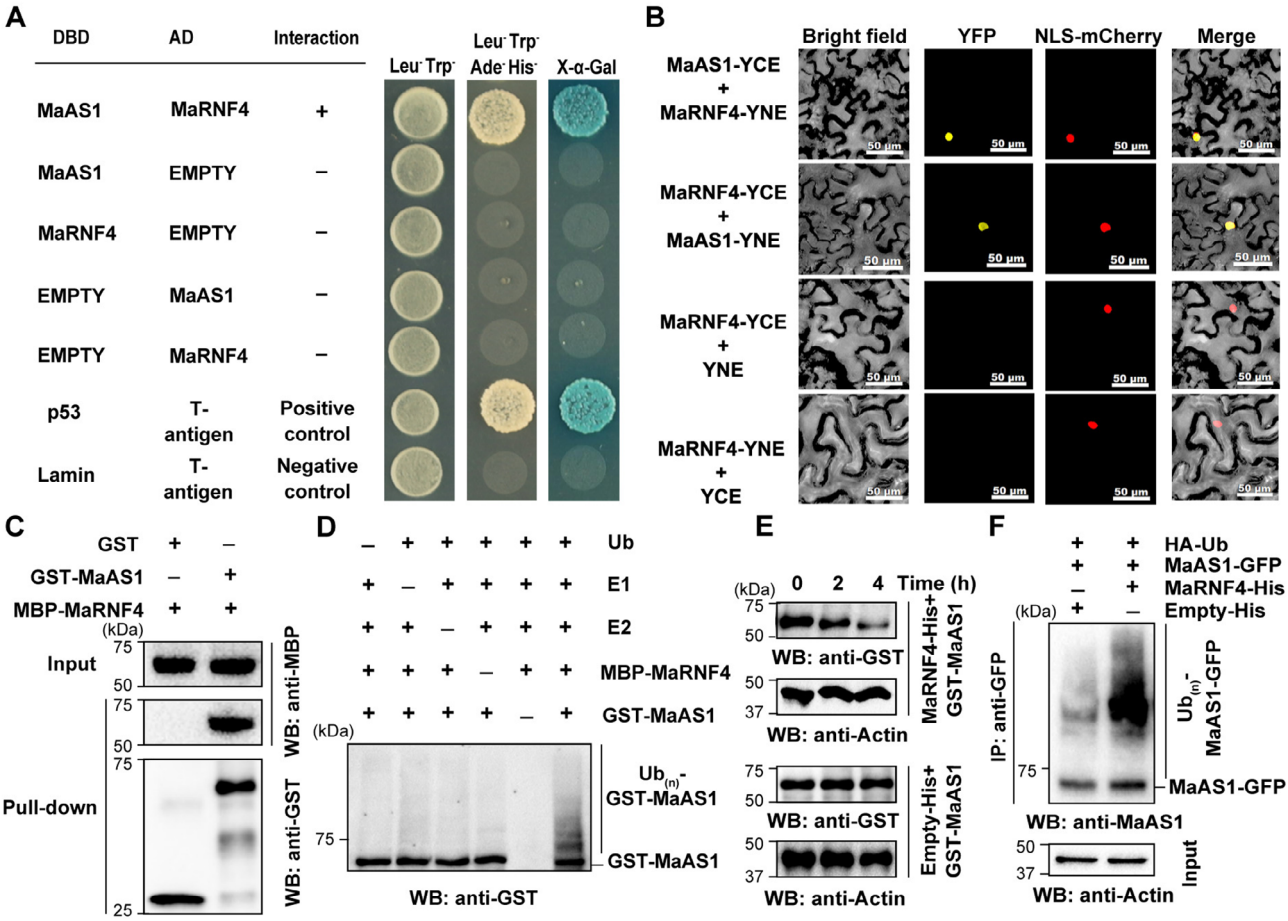
MaAS1 Interacting with MaRNF4 in vitro and in vivo. (A) Y2H and BiFC (B) assays showing the interaction between MaAS1 and MaRNF4. (C) GST pull‐down assay demonstrating the interaction between MaAS1 and MaRNF4 in vitro. (D) MaRNF4 ubiquitinating MaAS1 in vitro. Purified recombinant protein MBP‐MaRNF4 was incubated with GST‐MaAS1 in the presence or absence of Ub, E1 Ub‐activating enzyme (E1), and E2 Ub‐conjugating enzyme (E2). MaAS1 ubiquitination was detected via Western blot using an anti‐GST antibody. (E) Cell‐free degradation assay of recombinant MaAS1 protein. Crude proteins from tobacco leaves transiently expressing MaRNF4‐His or empty‐His were incubated with purified GST‐MaAS1 for the indicated time. GST‐MaAS1 protein levels were detected via Western blot using anti‐GST antibody, with Actin serving as a loading control. (F) MaRNF4 ubiquitinating MaAS1 in vivo. HA‐Ub and the recombinant protein MaAS1‐GFP were co‐infiltrated into tobacco leaves with MaRNF4‐His or empty‐His. Ubiquitinated MaAS1 was detected using an anti‐MaAS1 antibody, with Actin serving as a loading control. ‘−’ and ‘+’ denote no component and standard dosage, respectively.

Transient overexpression of *MaRNF4* in banana fruit significantly accelerated ripening (Figure 9A,B), leading to increased fruit softening and ethylene release (Figure 9C). This process was accompanied by an upregulation of *MaPME23*, *MaACS1* and *MaEXPA15* expression (Figure 9D). Similar effects were observed in tomato with stable *MaRNF4* overexpression, which also advanced the ethylene peak, accelerated softening, and upregulated *SlPME2.1*, *SlACS1B* and *SlEXP1* expression (Figure 9F–I). Notably, MaAS1 protein levels were reduced in *MaRNF4*‐overexpressing fruit compared with the control (Figure 9E). These findings indicated that MaRNF4 promoted the degradation of MaAS1 through ubiquitination, thereby relieving transcriptional repression of ripening‐related genes and accelerating fruit ripening.

**FIGURE 9:**
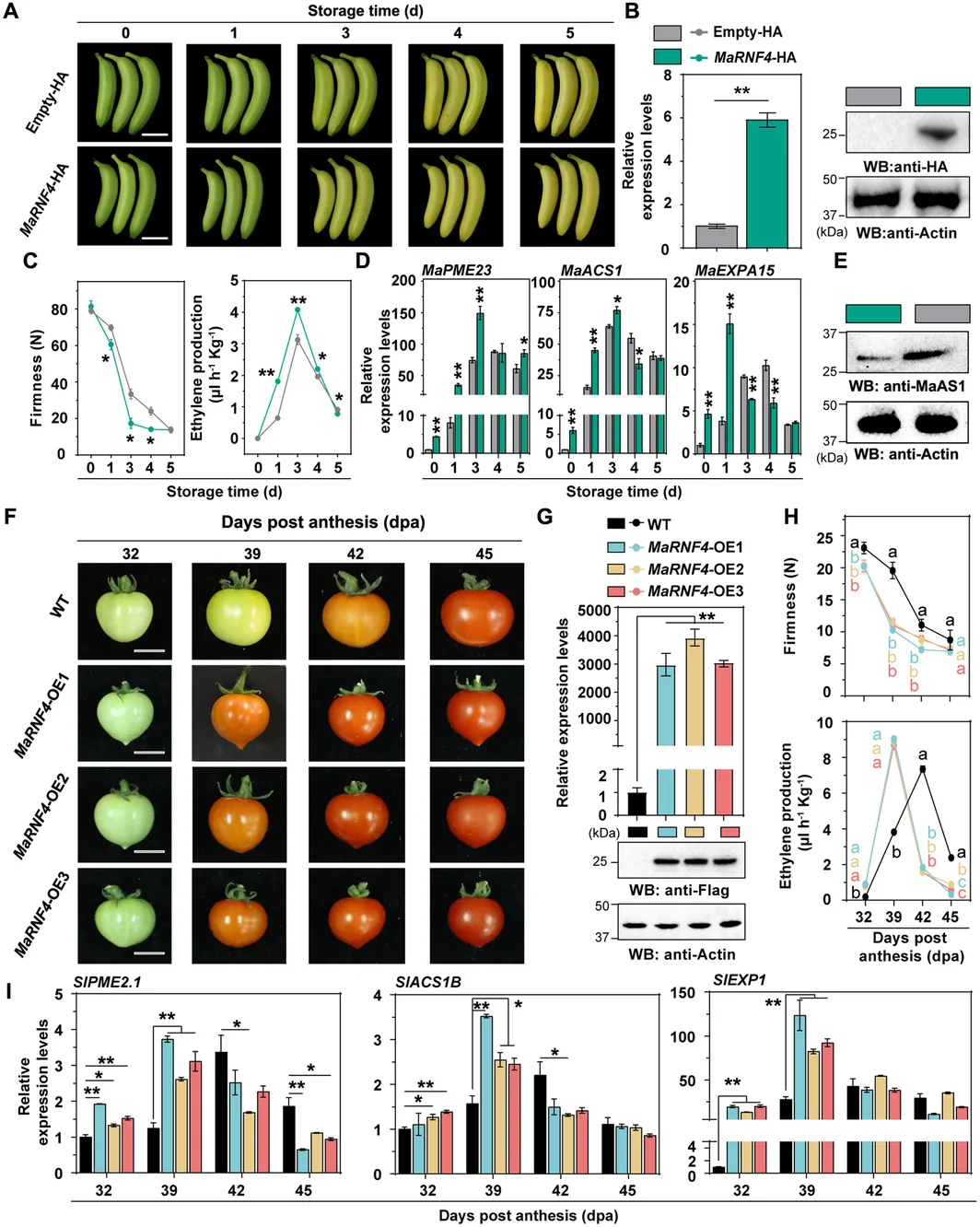
Functional analysis of *MaRNF4* in banana and tomato fruit. (A) Phenotypic variations among banana fruits transiently overexpressing *MaRNF4*‐HA or empty‐HA. Images were digitally extracted. Scale bar = 5 cm. (B) The overexpression of *MaRNF4* in banana fruit was detected via RT‐qPCR and Western blot using anti‐HA antibody. (C) Changes in firmness and ethylene production in banana fruits injected with empty‐HA or *MaRNF4*‐HA. (D) Relative mRNA abundance of *MaPME23*, *MaACS1* and *MaEXPA15* in *MaRNF4*‐HA and empty‐HA banana fruit during ripening, as determined via RT‐qPCR. (E) Detection of MaAS1 protein accumulation in banana pulp transiently overexpressing *MaRNF4*. (F) Tomato fruit images of WT and three *MaRNF4*‐OE lines at 32, 39, 42 and 45 dpa are shown. Bar, 1 cm. (G) Overexpression of *MaRNF4* was detected by RT‐qPCR and Western blot, with total protein and mRNA extracted at 45 dpa. The RT‐qPCR data were expressed as a ratio to WT and presented as mean ± SE (*n* = 3). (H) Changes in fruit firmness and ethylene production at different time point were observed with data presented as mean ± SE (*n* = 3). The different letters denote a significant difference (one‐way ANOVA, *p* < 0.05). (I) The expression level of *SlPME2.1*, *SlACS1B* and *SlEXP1* in WT and transgenic lines. The data were expressed as ratio relative to 32 dpa of WT and presented as mean ± SE (*n* = 3). The * indicates a significant different (Student's *t*‐test, *p < 0.05, **p < 0.01).

### 2.6 Phosphorylation by MaMPK3‐2 Counteracting MaRNF4‐Mediated Ubiquitination of MaAS1 to Delay Banana Fruit Ripening

Emerging evidence indicates that various PTMs interact to regulate TF function in plants (Lu et al. 2011; Zhang et al. 2025). In the present study, MaMPK3‐2‐mediated phosphorylation stabilized MaAS1, whereas MaRNF4‐mediated ubiquitination promoted its degradation, implying an antagonistic relationship between these modifications. To dissect the molecular mechanism underlying MaAS1 regulation by the two modifications, transient overexpression of *MaMPK3‐2* was performed in banana fruit. Phenotypic assays demonstrated that ectopic *MaMPK3‐2* overexpression postponed fruit ripening (Figure 10A,B), reduced fruit softening and ethylene release (Figure 10C), and downregulated MaAS1 target genes (Figure 10D). Immunoprecipitation and Western blot analysis revealed that *MaMPK3‐2* overexpression decreased MaAS1 ubiquitination (Figure 10E), indicating that phosphorylation inhibited its degradation. Phosphorylation at specific sites (Ser61, Thr92 and Ser120) was observed to directly counteract MaRNF4‐dependent ubiquitination of MaAS1, as demonstrated by reduced ubiquitination and increased protein accumulation in the phosphomimetic mutant compared with the nonphosphorylatable mutant (Figure 10F). Collectively, the MaMPK3‐2‐MaAS1‐MaRNF4 regulatory module modulates banana fruit ripening via antagonistic phosphorylation and ubiquitination of MaAS1 (Figure 10G).

**FIGURE 10:**
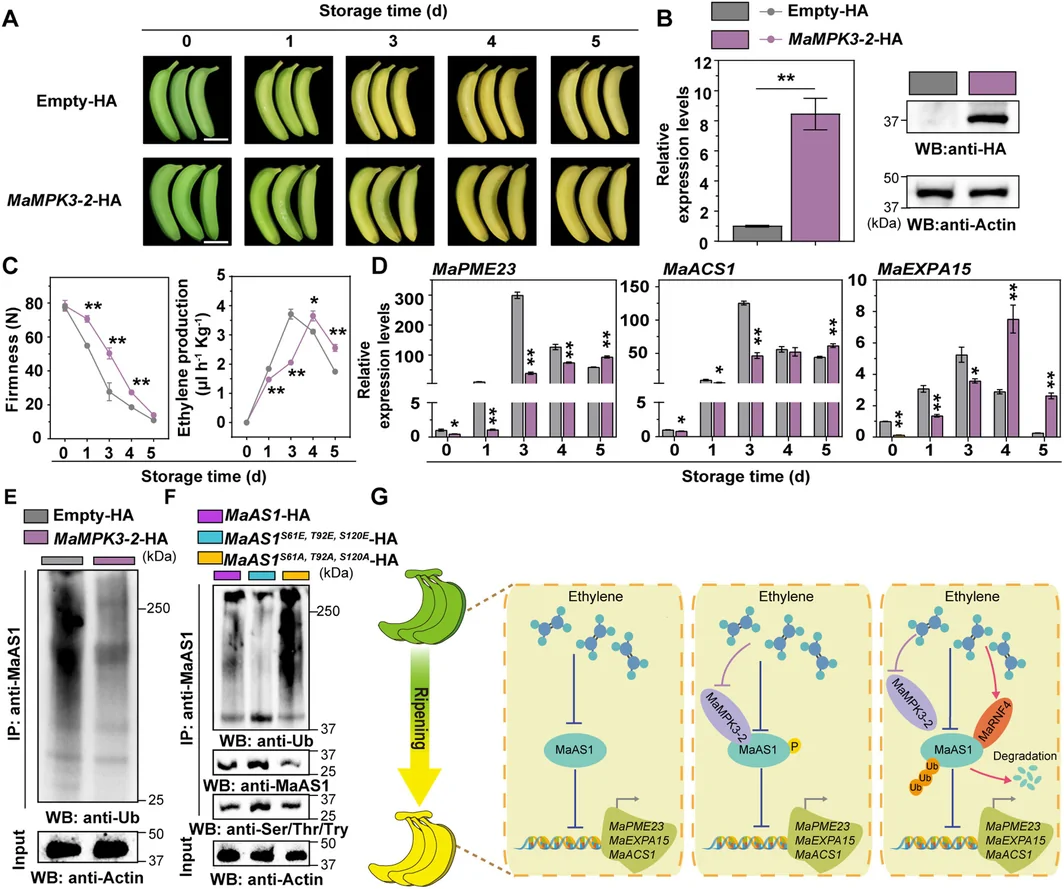
Functional analysis of *MaMPK3‐2* in banana fruit. (A) Phenotypic variations among banana fruits transiently overexpressing *MaMPK3‐2*‐HA or empty‐HA. Images were digitally extracted. Scale bar = 5 cm. (B) The overexpression of *MaMPK3‐2* in banana fruit was detected via RT‐qPCR and Western blot. (C) Changes in firmness and ethylene production among banana fruits injected with empty‐HA or *MaMPK3‐2*‐HA. (D) Relative mRNA abundance of *MaPME23*, *MaACS1* and *MaEXPA15* in *MaMPK3‐2*‐overexpression and empty‐HA banana fruit during ripening, as determined via RT‐qPCR. Data are presented as mean ± SE (*n* = 3 biological replicates). *Indicates a significant difference (Student's *t*‐test, *p < 0.05, **p < 0.01). (E) Detection of MaAS1 ubiquitination levels in banana pulp transiently overexpressing *MaMPK3‐2* via Western blot analysis. (F) Western blot analysis of MaAS1 protein accumulation, ubiquitination and phosphorylation levels in banana pulp transiently overexpressing *MaAS1*, *MaAS1* ^*S61E, T92E, S120E*^ and *MaAS1* ^*S61A, T92A, S120A*^. (G) Proposed model illustrating phosphorylation‐ubiquitination crosstalk regulating MaAS1 during banana fruit ripening. MaMPK3‐2 phosphorylates MaAS1 at Ser61, Thr92 and Ser120, enhancing its transcriptional repression activity and protein stability, thereby preventing its ubiquitin‐mediated degradation by MaRNF4 and further regulating banana fruit ripening.

## 3 Discussion

In this study, we identified MaAS1 as a ripening‐repressive R2R3‐MYB TF and elucidated how its stability and activity were regulated through a phosphorylation‐ubiquitination mechanism involving the MaMPK3‐2‐MaAS1‐MaRNF4 regulatory module (Figure 10G). R2R3‐MYB TFs play crucial roles in fruit ripening, as demonstrated in previous studies (Yan et al. 2020; Zhang et al. 2024). Our previous research identified MaMYB44 and MaMYB60 as ripening repressors in banana (Wei, Yang, et al. 2023; Luo et al. 2025). This study extended the paradigm by demonstrating how a repressive MYB TF was regulated at the post‐translational level.

Fruit ripening involves changes in ethylene production and cell wall disassembly (Xiao et al. 2018; Kuang et al. 2021). Various TF families, including NAC, bHLH and ERF, regulate banana fruit ripening by controlling ethylene production and fruit softening (Xiao et al. 2018; Shan et al. 2020; Xie et al. 2026). In this study, we employed DAP‐seq to identify *MaACS1*, *MaPME23* and *MaEXPA15* as direct targets of MaAS1. Furthermore, a unique binding motif, RMAACATBYT, was also identified (Figure 2). This motif differed from that recognized by the *Arabidopsis* ortholog AtAS1 (Guo et al. 2008), indicating species specific DNA‐binding properties in banana. MaAS1 suppressed these target genes through this motif, and overexpression of *MaAS1* reduced the expression of their tomato orthologs, resulting in delayed ripening (Figures 6 and 7).

MAPK cascades modulate the activity of key TFs during the ripening transition. SlWRKY6 and MdWRKY9 are phosphorylated by MAPKs and play a role in fruit ripening regulation (Wang et al. 2025). In banana, regulators such as MabZIP21 and MaMYB4 are also phosphorylated, ultimately associating MAPK signalling with fruit ripening (Wu et al. 2022; Yang et al. 2023). Our analysis revealed that the group A kinase MaMPK3‐2 interacted with and phosphorylated MaAS1 at Ser61, Thr92 and Ser120 (Figures 4 and 5). Phosphorylation can affect protein function by altering subcellular localization, DNA‐binding capacity, transcriptional activity, or protein stability (Kim et al. 2017). Using phosphomimetic and nonphosphorylatable mutants, we demonstrated that phosphorylation of MaAS1 enhanced its transcriptional repression activity and increased its protein abundance (Figure 6). The nonphosphorylatable mutant behaved similarly to the empty vector in banana but accelerated ripening in tomato, potentially owing to the expansion of target gene networks that amplify downstream signalling output (Figures 6 and 7). This finding was consistent with the observation that homologous target genes of MaMYB73 exhibited an expansion effect in tomato (Luo et al. 2026), highlighting the importance of these three phosphorylation sites in MaAS1 function. Therefore, MaMPK3‐2 acted as a ripening repressor by phosphorylating MaAS1, similar to the MaMPK14‐MaMYB4 phosphorylation module previously reported (Yang et al. 2023).

Ubiquitin‐dependent degradation plays a key role in regulating the levels of ripening regulators (Wei, Yang, et al. 2023; Li, Liu, et al. 2025). For example, the negative regulator of fruit ripening, SlSAD8, interacts with the E3 ubiquitin ligase SlAP1, disrupting the degradation pathway of chloroplast‐associated proteins and leading to increased chloroplast protein levels (Xu et al. 2025). In this study, we identified MaRNF4 as a RING‐type E3 ligase that was activated during ripening and targeted MaAS1 for degradation. This degradation of MaAS1 mitigated its repression of *MaACS1*, *MaEXPA15* and *MaPME23*, ultimately accelerating fruit ripening (Figures 8 and 9). Similarly, the ethylene‐activated E3 ligase MdEAEL1 ubiquitinates and degrades the transcriptional repressor MdZFP3, which otherwise forms a repression complex to suppress cell wall degradation‐related genes, thereby promoting apple fruit softening (Li, Liu, et al. 2025). These findings supported the idea that E3 ligases act as positive regulators of climacteric fruit ripening by removing inhibitory factors.

Phosphorylation and ubiquitination often interact in plant signalling pathways, either antagonistically or cooperatively (Wei, Liu, et al. 2023; Wang et al. 2024). For example, OsCPK4‐mediated phosphorylation promotes OsRING142‐dependent ubiquitination of OsRbohB (Tao et al. 2025), whereas phosphorylated MaMYB4 is less susceptible to ubiquitination (Yang et al. 2023). Our study revealed that MaMPK3‐2‐mediated phosphorylation counteracted MaRNF4‐dependent ubiquitination of MaAS1. Overexpression of *MaMPK3‐2* reduced MaAS1 ubiquitination and increased its phosphorylation and protein levels, and the phosphomimetic MaAS1 mutant exhibited decreased ubiquitination compared with the nonphosphorylatable mutant (Figure 10). These results indicated that phosphorylation at Ser61, Thr92 and Ser120 protected MaAS1 from degradation, thereby stabilizing the repressor and delaying ripening. In contrast, phosphorylation of the transcriptional repressor FvMYB44s reduces its stability (Feng et al. 2026). This divergence possibly stems from differences in kinase specificity and the structural effects of phosphorylation at specific sites, which can influence protein conformation and interactions. Understanding how phosphorylation influences TF architecture to regulate protein stability represents a key area for future research.

In summary, our data indicated that MaAS1 levels were regulated by a balance between phosphorylation and ubiquitination. In unripe fruit, MaMPK3‐2 phosphorylated MaAS1, maintaining its stability and repressive function to delay ripening. As ripening progressed, *MaMPK3‐2* expression decreased, whereas the levels of the E3 ligase, MaRNF4, increased, leading to MaAS1 ubiquitination and degradation. This condition resulted in the activation of ripening‐related genes such as *MaACS1*, *MaPME23* and *MaEXPA15*, promoting ethylene production and cell wall disassembly (Figure 10). This phosphorylation‐ubiquitination switch is not limited to banana. In apple, the E3 ligase MdPUB24 ubiquitinates the transcriptional repressor MdNAC72, promoting its degradation via the 26S proteasome and thereby relieving the repression of the cell wall degradation‐related gene *MdPG1* to promote fruit softening (Wei, Liu, et al. 2023). Notably, ethylene‐induced phosphorylation of MdNAC72 by MdMAPK3 increases, rather than protects against, its ubiquitination. This contrasts with the antagonistic mode we observed for MaAS1, where phosphorylation directly counteracts ubiquitination. In tomato, the sugar transporter SlSWEET16 is co‐regulated by phosphorylation and ubiquitination at its C‐terminus: phosphorylation by SlSnRK2.3 and SlSnRK2.4 enhances its stability, whereas ubiquitination by the E3 ligase SlTT3.1 L2 promotes its degradation. Together, these processes coordinate fruit sugar accumulation (Song et al. 2026), suggesting that the interplay between phosphorylation and ubiquitination represents a conserved strategy for fine‐tuning ripening regulators across climacteric fruits. However, the specific kinase‐substrate pairs and regulatory outcomes may vary. These findings reveal a phosphorylation‐ubiquitination regulatory module that controls the abundance of a key ripening repressor. The manipulation through either genetic stabilization of MaAS1 or targeted disruption of its ubiquitination would offer a tangible molecular strategy to extend postharvest shelf life while preserving fruit quality in future application.

## 4 Materials and Methods

### 4.1 Plant Materials and Treatments

Banana fruit ( *Musa acuminata* AAA group, Cavendish subgroup) at 70%–80% commercial maturity was harvested from a commercial farm in Chongzuo, Guangxi, China. Individual fingers were separated, and uniform, undamaged samples were divided into two groups (100 fingers each): natural ripening (untreated control) and ethylene treatment (100 μL L^−1^ ethylene for 18 h). Both groups were stored at 22°C and 90% relative humidity (RH) for 7 days. Fruit firmness and ethylene production were measured at 0, 1, 3, 5 and 7 days post‐treatment, with 3–6 biological replicates per time point. All samples were flash‐frozen in liquid nitrogen and stored at −80°C.

Tobacco (*Nicotiana benthamiana*) plants were grown at 22°C under long‐day conditions (16‐h light/8‐h dark). Four‐ to six‐week‐old plants were used for transient assays.

Tomato ( *Solanum lycopersicum* cv. Micro‐Tom) plants were grown under controlled conditions at 25°C/20°C (day/night), 60% RH, a light intensity of 250 μmol m^−2^ s^−1^, and a 14‐h light/10‐h dark photoperiod. Fruit firmness and ethylene production were measured in T2 transgenic lines and wild‐type controls at four ripening stages: mature green, breaker, orange and red.

### 4.2 Physiological Measurements

Fruit firmness was measured using a GY‐4 digital hardness tester with a 10 mm probe for banana pulp and a 3.5 mm probe for tomato fruit. Ethylene production was measured by sealing three fruits in a container for each of the three biological replicates. After incubation at 22°C ± 1°C for 3 h, 1 mL of headspace gas was collected and analysed via gas chromatography (GC‐17A; Shimadzu, Japan) using an activated alumina column (200 cm × 0.3 cm) and a flame ionization detector.

### 4.3 Sequence Alignment and Phylogenetic Analysis

Amino acid sequences were extracted from the banana genome database (https://banana‐genome‐hub.southgreen.fr/), *Arabidopsis* genome database (https://www.arabidopsis.org/), tomato genome database (https://solgenomics.net/) and rice genome database (https://riceome.hzau.edu.cn/). The sequences were aligned using ClustalW and visualized with Jalview. Phylogenetic trees were constructed via the neighbour‐joining method in MEGA12 software with 1000 bootstrap replicates.

### 4.4 RT‐qPCR Assay

Total RNA was extracted from banana and tomato using the polysaccharide‐polyphenol plant RNA kit (TIANGEN, China). cDNA synthesis was performed using HiScript II Q RT SuperMix (+gDNA wiper) (Vazyme, China). Quantitative PCR was performed on a Bio‐Rad CFX96 system using SYBR Green PCR Mix (Yeasen), with *MaRPS4* as the reference gene (Chen et al. 2011). Primer sequences are listed in Table S1.

### 4.5 Subcellular Localization

The coding sequence (CDS) of *MaAS1* (excluding the stop codon) was inserted into the pBE‐GFP vector. pBE‐*MaAS1*‐GFP or empty pBE‐GFP was co‐infiltrated with a nuclear localization signal (NLS)‐mCherry construct into *Nicotiana benthamiana* leaves. Fluorescence was observed 36–48 h post‐infiltration using a Zeiss Axioskop 2 Plus fluorescence microscope. GFP fluorescence was detected using a 488 nm excitation wavelength and a 505–530 nm emission filter, whereas mCherry fluorescence was detected using a 594 nm excitation wavelength and a 610–650 nm emission filter.

### 4.6 Dual‐Luciferase Reporter and Transcriptional Activity Assays in Tobacco

Dual‐luciferase reporter assay was performed following the protocol outlined by Hellens et al. (2005), with minor modifications. The CDS of *MaAS1*, *MaAS1* ^*S61E, T92E, S120E*^ and *MaAS1* ^*S61A, T92A, S120A*^ were cloned into the effector vector pGreenII 62SK‐BD. These constructs, along with 62SK‐BD‐VP6 (positive control) and 62SK‐BD (negative control), were used as effectors for transcriptional activity analysis. The reporter construct contains LUC and REN coding genes as well as 5×GAL4 driven by the CaMV35S promoter. Full‐length *MaAS1* was also cloned into pGreenII 62‐SK as an effector. The promoters of *MaPME23*, *MaACS1* and *MaEXPA15* were inserted into the reporter vector pGreenII 0800. The effector and reporter constructs were transformed into *A. tumefaciens* strain EHA105, respectively and then co‐infiltrated into leaves of 4–6‐week‐old *N*. *benthamiana* via *Agrobacterium*‐mediated infiltration. Dual‐luciferase activity was measured 48–60 h post‐infiltration using the DLR gene assay kit (Yeasen, 11402ES60) and a Luminoskan Ascent Microplate Luminometer (Thermo Fisher Scientific) according to the manufacturer's protocol. All results are presented as the ratio of LUC to REN. Each assay included at least three biological replicates.

### 4.7 Yeast Transcriptional Activity and Two‐Hybrid Assays

For yeast transcriptional activity assays, the CDS of *MaAS1* was inserted into the pGBKT7 (BD) vector. The resulting plasmid was introduced into the Y2H yeast strain via the lithium acetate method and cultured on SD/−Trp medium at 30°C for 3 days. Afterward, transformants were streaked onto SD/−Trp/−Ade/–His medium to evaluate transcriptional activity based on colony growth and α‐galactosidase activity.

For the yeast two‐hybrid (Y2H) assay, the CDS of *MaAS1* and *MaRNF4* were inserted into the pGBKT7(BD) (bait) and pGADT7(AD) (prey) vectors, respectively. The plasmid combinations were co‐transformed into yeast cells and cultured on SD/−Leu/−Trp medium at 30°C for 3 days. Subsequently, the transformed cells were transferred to SD/−Leu/−Trp/−Ade/–His medium to screen for protein–protein interactions. Interactions were evaluated based on colony growth and α‐galactosidase activity.

### 4.8 BiFC Assay

The following genes were separately cloned into pUC‐pSPYNE or pUC‐pSPYCE vectors: *MaAS1*, *MaMPK3‐2*, *MaMPK9‐1*, *MaMPK9‐4*, *MaMPK11‐2*, *MaMPK11‐3*, *MaMPK20‐4* and *MaRNF4*. The resulting constructs were transformed into *Agrobacterium tumefaciens* strain EHA105 and infiltrated into tobacco leaves. NLS‐mCherry was used as a nuclear marker, and single‐gene infiltrations served as negative controls. YFP fluorescence signals were observed 48 h post‐infiltration using a fluorescence microscope (Zeiss Axioskop 2 plus, Carl Zeiss Microscopy, GmbH).

### 4.9 Protein Expression and Pull‐Down Assay

*MaAS1*, *MaAS1* ^*S61E, T92E, S120E*^ and *MaAS1* ^*S61A, T92A, S120A*^ were inserted into the GST‐tagged vector pGEX‐4T. MaMPK3‐2 and MaRNF4 were cloned into the MBP‐tagged vector pMAL‐c2X. The recombinant proteins were expressed in Rosetta (DE3) cells. GST‐ and MBP‐fusion proteins were purified using the Pierce GST spin purification kit (Yeasen, Shanghai, China) and MBP‐Trap affinity resin (New England Biolabs, Beverly, MA, USA), respectively, according to the manufacturer's instructions.

Pull‐down assays were performed following the protocol outlined by Einarson et al. (2007), with minor modifications. The eluted proteins were separated via SDS‐PAGE and analysed via Western blotting with anti‐GST (Zen‐bio, China) and anti‐MBP (Zen‐bio, China) primary antibodies. Subsequently, a goat anti‐rabbit IgG peroxidase secondary antibody (Thermo Scientific) was applied. Signals were detected using the chemiluminescent substrate SuperSignal West Pico (Thermo Scientific), and images were captured using a ChemiDoc MP Imaging System (Bio‐Rad, Hercules, CA, USA).

### 4.10 DAP‐Seq

DAP‐seq was performed following the protocol outlined by Bartlett et al. (2017), with minor modifications. Three biological replicates were included. A list of MaAS1 binding peaks across all replicates is provided in Data S4. The procedure included the following steps: (1) DNA fragmentation and library construction, (2) protein expression, (3) DNA‐protein binding with Western blot validation, (4) sequencing and (5) sequencing data analysis. Genomic DNA extraction, library preparation and sequencing were conducted by Jige Biotechnology Co. Ltd. (Guangzhou, China), whereas primary data analysis was performed by Yongji Biotechnology Co. Ltd. (Guangzhou, China).

For bioinformatic analysis of the sequencing data, the HOMER software program was used to identify the binding motifs of MaAS1 (Heinz et al. 2010). Based on these identified motifs, GEM (Genome Wide Binding Event Finding and Motif Discovery) was used to predict the genome‐wide cis‐acting elements bound by MaAS1. GO enrichment analysis was performed using the Gene Ontology database (http://geneontology.org/), with a screening threshold of *p* < 0.05.

### 4.11 EMSA

To analyse binding to the RMAACATBYT element, biotin‐labelled promoter fragments from *MaPME23*, *MaACS1* and *MaEXPA15* were used in EMSA. A 50× cold probe was used as a competitor, and a mutated element (AAAAAAAAAA) served as a specific competition probe. The assay was performed using an EMSA/Gel‐Shift kit (Beyotime, Shanghai, China), with GST used as a negative control. Biotin signals were detected using a ChemiDoc MP Imaging System (Bio‐Rad, Hercules, CA, USA).

### 4.12 ChIP‐qPCR Assay

ChIP‐qPCR assays were conducted according to the protocol reported by Han et al. (2016). Briefly, banana pulp tissues were fixed with 1% formaldehyde to cross‐link the genomic DNA and associated proteins. The isolated chromatin was fragmented via sonication to an average length of 500 base pairs. Immunoprecipitation was performed using an anti‐MaAS1 antibody custom produced by HuaAn Biotechnology, whereas IgG was used as an internal negative control to evaluate enrichment signals in ChIP‐qPCR. All experiments were carried out with three biological replicates.

### 4.13 In Vitro Phosphorylation Assay

MBP‐MaMPK3‐2 (1 μg) and GST‐MaAS1 (3 μg) were incubated in kinase buffer (20 mM Tris–HCl, 100 mM NaCl, 20 mM MgCl_2_, 2 mM DTT and 10 mM ATP) at 37°C for 30 min. For the AP reaction, 1 μL of Fast Alkaline Phosphatase (Thermo Fisher) was added to the reaction mixture. Subsequently, proteins were separated via SDS‐PAGE (10% w/v) with 0.1 mM MnCl_2_ and 0.1 mM Phos‐tag (Wako) and identified via immunoblotting using an anti‐GST antibody.

### 4.14 LC–MS/MS Assay

Phosphorylated proteins from in vitro phosphorylation assays were separated via SDS‐PAGE (10% w/v) and stained with Coomassie Brilliant Blue R‐250. The target bands were excised and analysed via liquid chromatography–tandem mass spectrometry (LC–MS/MS) at PTM BIO (Hangzhou, China).

### 4.15 Transient Overexpression in Banana and Stable Overexpression Analysis in Tomato

To transiently overexpress genes in banana, *MaAS1*, *MaAS1* ^*S61E, T92E, S120E*^, *MaAS1* ^*S61A, T92A, S120A*^, *MaMPK3‐2* and *MaRNF4* were inserted into the pCXUN‐HA vector and transformed into *Agrobacterium* strain EHA105. The recombinant plasmids and an empty pCXUN‐HA vector were infiltrated into banana pulp according to the method described by Yang et al. (2023). After a 24‐h incubation period, the fruits were treated with 100 μL·L^−1^ ethylene for 16 h and then stored at 22°C (90% RH) for 5 d. Ethylene production, fruit firmness and gene expression were assessed using three biological replicates.

To investigate stable overexpression in tomato, the same gene set was cloned into the pBI121‐FLAG plasmid and then transformed into *A. tumefaciens* strain GV3101. Transgenic Micro‐Tom tomato plants were created according to the method described by Fillatti et al. (1987). Fruit firmness and ethylene release were measured in three independent T2 transgenic lines.

### 4.16 In Vitro and In Vivo Ubiquitination Assay

In vitro ubiquitination assays were performed following the protocol described by Shan et al. (2020). GST‐MaAS1 was incubated with or without E1 (Boston Biochem), E2 (Boston Biochem), ubiquitin (Ub, Boston Biochem) and the MBP‐MaRNF4 fusion protein. The reaction mixtures were separated via SDS‐PAGE and probed using an anti‐GST antibody (Zen‐bio, China).

To investigate in vivo ubiquitination, MaAS1‐pEAQ‐GFP and MaRNF4‐pEAQ‐His were co‐expressed in tobacco leaves through *Agrobacterium* infiltration. After 36 h, 10 μM MG132 was added, and samples were incubated for an additional 12 h. Crude protein extracts were immunoprecipitated using an anti‐GFP antibody (Zen‐bio, China), separated via SDS‐PAGE, and analysed via immunoblotting with an anti‐MaAS1 antibody.

### 4.17 Cell‐Free Degradation Assay

Crude extracts from tobacco leaves expressing MaRNF4‐pEAQ‐His (Shan et al. 2020) were incubated with GST‐MaAS1 at 22°C for 0, 2 or 4 h. Afterward, the samples were separated via SDS‐PAGE and analysed via immunoblotting with anti‐GST (Zen‐bio, China) and anti‐Actin antibodies.

### 4.18 Co‐Immunoprecipitation From Banana Fruit

Total proteins were extracted from empty‐HA, *MaMPK3‐2*‐HA, *MaAS1*‐HA, *MaAS1* ^*S61E, T92E, S120E*^‐HA and *MaAS1* ^*S61A, T92A, S120A*^‐HA samples. Anti‐MaAS1 antibody was added to the extracts and incubated overnight at 4°C. Afterward, protein A beads were added and incubated, with simultaneous shaking for 5 h at 4°C. The supernatant was obtained via centrifugation and used for immunoblot detection with anti‐Actin, anti‐Ub (Sigma, Germany), anti‐MaAS1 (custom produced by HuaAn Biotechnology) and anti‐Ser/Thr/Tyr (Abcam, England) antibodies.

### 4.19 Data Analysis

Statistical significance was determined via Student's *t*‐test, with * (*p* < 0.05) and ** (*p* < 0.01) indicating significant differences. Multiple comparisons were performed via one‐way ANOVA, with lowercase letters indicating significant differences at *p* < 0.05.

### 4.20 Accession Numbers

Sequence data from this article can be found in the banana genome database (https://banana‐genome‐hub.southgreen.fr/), tomato genome database (https://solgenomics.sgn.cornell.edu/), *Arabidopsis* genome database (https://www.arabidopsis.org/) and rice genome database (https://riceome.hzau.edu.cn/) under the following accession numbers: *MaAS1* (Ma05_g031440), *MaMPK3‐2* (Ma09_04230), *MaRNF4* (Ma08_00800), *MaACS1* (Ma04_g35640), *MaEXPA15* (Ma06_g12190), *MaPME23* (Ma07_g15530), *SlEXP1* (Solyc06g051800), *SlACS1B* (Solyc08g081540), *SlPME2.1* (Solyc07g064180), *AtMYB91* (AT2G37630), *AtMPK3* (At3g45640), *AtMPK6* (At2g43790), *AtMPK10* (At3g59790), *OsMYB91* (LOC_Os12g38400) and *SlMYB91* (Solyc09g010840).

## Author Contributions

W.‐J.L. conceived and designed the study. T.‐T.L., Y.‐Y.Y. and H.‐K.T. performed the experiments and data analysis. T.‐T.L. wrote the manuscript. Y.‐Y.Y., W.W., W.S., J.‐F.K. and J.‐Y.C. provided valuable advice on the manuscript. Y.‐Y.Y., J.‐F.K., J.‐Y.C. and W.‐J.L. revised the manuscript. All authors contributed to the article and approved the submitted version.

## Funding

This work was supported by the National Key R&D Program of China (grant no. 2022YFD2100100) and the National Natural Science Foundation of China (grant no. 31830071).

## Conflicts of Interest

The authors declare no conflicts of interest.

## Supporting information


**Data S1:** DEGs in unripe and ripe banana.
**Data S2:** Genome‐wide identification of MaAS1 target genes.
**Data S3:** Expression level of *MaMPKs* in transcriptome data.
**Data S4:** List of MaAS1‐binding peaks.

**Figure S1:** Amino acid analysis of MaAS1 with similar proteins from other plants.
**Figure S2:** Pearson correlation plot (A) and correlation scatter plot (B) of MaAS1‐DAP‐Seq.
**Figure S3:** BiFC assay. MaAS1 does not interact with other MaMPKs except MaMPK3‐2. Bar, 50 μm.
**Figure S4:** Phylogenetic analysis and amino acid sequence alignment of MaMPK3‐2.
**Figure S5:** Expression characteristics and ubiquitin ligase activity assays of MaRNF4.
**Table S1:** Summary of primers used in the study.

## Data Availability

Data will be made available on request.
