# Supplementary material for: MaMPK3‐2 Phosphorylating and Stabilizing Transcriptional Repressor MaAS1 by Counteracting MaRNF4‐Mediated Ubiquitination During Banana Fruit Ripening
Source: Plant Biotechnol J. 2026 Oct 13:10.1111/pbi.70769. Online ahead of print. doi: 10.1111/pbi.70769 (PMC13660451; doi:10.1111/pbi.70769)
Supplement: Supplementary file 1 [file PBI-9999-0-s001.docx]

**Supporting information**

**Figure S1.** Amino acid analysis of MaAS1 with similar proteins from other plants. The protein sequence of MaAS1 was compared with AtMYB91 (At2g37630.1), SlMYB91 (Solyc09g01840.1.1) and OsMYB91 (LOC_Os12g38400.1) by ClustalW. Identical and similar amino acid were marked by blue and light blue shade.

**Figure S2.** Pearson correlation plot **(A)** and correlation scatter plot **(B)** of MaAS1-DAP-Seq.

**Figure S3.** BiFC assay. MaAS1 does not interact with other MaMPKs except MaMPK3-2. Bar, 50 μm.

**Figure S4.** Phylogenetic analysis and amino acid sequence alignment of MaMPK3-2. **(A)** Phylogenic relationship analysis of MaMPK3-2. The protein sequence of MaMPK3-2 was compared with MPKs from *Arabidopsis thaliana*. Multiple sequence alignment was performed by MEGA 12. The phylogenic tree was constructed by MEGA 12 with neighbor-joining method was used (bootstrap replicates=1000). The MPKs were divided into four subgroups A-D, and their function were described in the annotations. (**B)** Amino acid alignment of MaMPK3-2 with AtMPK3 (At3g45640), AtMPK6 (At2g43790), AtMPK10 (At3g59790) from subgroup A. The alignment was performed by ClustalW. Identical and similar amino acids were presented by blue and light blue shade. The red rectangle indicates TEY activation motif.

**Figure S5.** Expression characteristics and ubiquitin ligase activity assays of MaRNF4. **(A)** Relative expression levels of *MaRNF4* in banana fruit during natural and ethylene-induced ripening. The data were presented as mean ± SE of three replicates, with different letters to indicating a significant difference (one-way ANOVA, *p* < 0.05). **(B)** Analysis of MaRNF4 autoubiquitination

**Table S1.** **Summary of primers used in the study**

| **Assay** | **Primer sequence (5’-3’)** | | **Restriction Site** |
| --- | --- | --- | --- |
| RT-qPCR | *MaRPS4-qF:* TGAGAGTGGCTTGACCCTGA  *MaAS1-qF:* AACTACCTCAAGCCGGGAAT  *MaRNF4-qF:* GGTTCCGAATTTGGATCTGA  *MaMPK3-2-qF:* CGAACGCTACGGGAGATCAA  *MaEXPA15-qF:* CAGCATTTCGATCTCTCTCAGC  *MaPME23-qF:* CAGGGCAGCTCCTTGAATAC  *MaACS1-qF:* ACAAGTTCAAGATCACCCAAGC  *SlACT-qF:* TGTCCCTATTTACGAGGGTTATGC  *SlEXP1-qF:* ATCACAGCTACCAATTTCTGCC  *SlPME2.1-qF:* GTGCTTGGATGAGCTTGATTCC  *SlACS1B-qF:* GAGTGGAGGAGCAACTGGT | *MaRPS4-qR:* GTGACATTTAGTCGTCTGCTGG  *MaAS1-qR:*  TTGAAGACCTCCCACCACTT  *MaRNF4-qR:* GTCACGACCTCATCCTCCAT  *MaMPK3-2-qR:* GCAGTGTAGTCCGTCGAGTT  *MaEXPA15-qR:* GAACCTGATCCCTCCTCTCTTC  *MaPME23-qR:* GTCACCTCCGAGAACTCCAG  *MaACS1-qR:* AGTGCATCCTTTTCTCGTTGAC  *SlACT-qR:* CAGTTAAATCACGACCAGCAAGAT  *SlEXP1-qR:* TGGGATCCTGCGATAAGTTACA  *SlPME2.1-qR:* CTTCATCATTTGGAGTTGTCACAG  *SlACS1B-qR:* GGTGTTGGGACCAAAAAGGC |  |
| Full length cloning | *MaAS1-F:* ATGAAGGATAGGCAGCGGTG  *MaAS1-R:* TCACCGCCCATGCGTCTC  *MaRNF4-F:* ATGAGCACCCTTGGACCATCAA  *MaRNF4-R:* CTACTTGGATGATGGAAGATACACAC  *MaMPK3-2-F:* ATGGACGGAGCTCCGGCGAAC  *MaMPK3-2-R:* TCAGCACTTGTAGTCTGGATTGAAC | |  |
| Subcellular localization | *MaAS1-pBE-GFP-F*: ATCTAGAGCAGTCGACGGTACCATGAAGGATAGGCAGCGGTG  *MaAS1-pBE-GFP-R*:  CTCCTCGCCCTTGCTCACCATCCGCCCATGCGTCTC | | *Sal* I  *Bam*H I |
| Y2H assay | *MaAS1-BD-F*: ATGGCCATGGAGGCCGAATTCATGAAGGATAGGCAGCGGTG  *MaAS1-BD-R*: TGCGGCCGCTGCAGGTCGACGTCACCGCCCATGCGTCTC  *MaAS1-AD-F*: GGAGGCCAGTGAATTCATGAAGGATAGGCAGCGGTG  *MaAS1-AD-R*: CGAGCTCGATGGATCCTCACCGCCCATGCGTCTC  *MaRNF4-AD-F*: GGAGGCCAGTGAATTCATGAGCACCCTTGGACCATCAA  *MaRNF4-AD-R*: CGAGCTCGATGGATCCCTACTTGGATGATGGAAGATACACAC | | *Eco*R I  *Sal* I  *Eco*R I  *Bam*H I  *Eco*R I  *Bam*H I |
| Protein expression and EMSA assay | *GST-MaAS1-F*: GGTTCCGCGTGGATCCATGAAGGATAGGCAGCGGTG  *GST-MaAS1-R*: AGTCACGATGCGGCCGCTCACCGCCCATGCGTCTC  *MBP-MaRNF4-F*: AGGGAAGGATTTCAGAATTCATGAGCACCCTTGGACCATCAA  *MBP-MaRNF4-R*: AGGTCGACTCTAGAGGATCCCTACTTGGATGATGGAAGATACACAC  *MBP-MaMPK3-2-F*: AGGGAAGGATTTCAGAATTCATGGACGGAGCTCCGGCGAAC  *MBP-MaMPK3-2-R*: AGGTCGACTCTAGAGGATCCTCAGCACTTGTAGTCTGGATTGAAC  *MaEXPA15-probe-F*: CAAAGTGCTCCTCGAGAATGTTTCCGGAGATGAGAATG  *MaEXPA15-probe-R*: CATTCTCATCTCCGGAAACATTCTCGAGGAGCACTTTG  *MaPME23-probe-F*: TGTGAGGACAGCTCAAAAATGTTGTGATGCCATCGTTTGA  *MaPME23-probe-R*: TCAAACGATGGCATCACAACATTTTTGAGCTGTCCTCACA  *MaACS1-probe-F*: GCGAACGAGATTATAAGAACAACATTCTACTTCAAATATTAATT  *MaACS1-probe-R*: AATTAATATTTGAAGTAGAATGTTGTTCTTATAATCTCGTTCGC | | *Bam*H I  *Not* I  *Eco*R I  *Bam*H I  *Eco*R I  *Bam*H I |
| ChIP-qPCR | *MaPME23-ChIP-F:* TGTGAGGACAGCTCAAAAATG  *MaPME23-ChIP-R:* TAAGCTCGTGGTAGGGTCAT  *MaEXPA15-ChIP-F:* TGTTGGCTCAATCATACGACA  *MaEXPA15-ChIP-R:* GCAGTCGATGATCCAAGAAGT  *MaACS1-ChIP-F:* GCTGGAAATGGACTGTTTGG  *MaACS1-ChIP-R:* GGGTGTAGTTTCCGAGTATCA | |  |
| Dual-luciferase expression assay | *62SK-BD-MaAS1-F*: CGCCGTCTAGAACTAGTGGATCCATGAAGGATAGGCAGCGGTG  *62SK-BD-MaAS1-R*: TCGATAAGCTTGATATCGAATTCTCACCGCCCATGCGTCTC  *62SK-MaAS1-F*: GGCCGCTCTAGAACTAGTGGATCCATGAAGGATAGGCAGCGGTG  *62SK-MaAS1-R*: ATCGATAAGCTTGATATCGAATTCCCGCCCATGCGTCTC  *MaEXPA15-0800-F*: TATAGGGCGAATTGGGTACCGTGGTTGGAGAAGGCATCAT  *MaEXPA15-0800-R*: TTGGCGTCTTCCATGGGAAAGAAGACGCCAACTGCT  *MaBAM3-0800-F*: TATAGGGCGAATTGGGTACCTTGGTGTGGAAGATTTCTTGG  *MaBAM3-0800-R*: TTGGCGTCTTCCATGGCGCGCCACTAAAAAGCC  *MaPME23-0800-F*: TATAGGGCGAATTGGGTACCAGGTTTTAGGATTTGCTATGATG  *MaPME23-0800-R*: TTGGCGTCTTCCATGGAGTCATTAATGGAGAGGTGATGA  *MaACS1-0800-F*: TATAGGGCGAATTGGGTACCCCAAGAAAGAAATGGGGATG  *MaACS1-0800-R*: TTGGCGTCTTCCATGGCCTCATGTGACCCGTTATCTC | | *Bam*H I  *Eco*R I  *Bam*H I  *Hin*d III  *Kpn* I  *Nco* I  *Kpn* I  *Nco* I  *Kpn* I  *Nco* I  *Kpn* I  *Nco* I |
| Transient overexpression | *pCXU-HA-MaAS1-F*: CATACGATGTTCCAGATTACGCTATGAAGGATAGGCAGCGGTG  *pCXU-HA-MaAS1-R*: TATTGCCAAATGTTTGAACGATCTCACCGCCCATGCGTCTC  *pCXU-HA-MaAS1^Ser61E, Thr92E, Ser120E^-F*: CATACGATGTTCCAGATTACGCTATGAAGGATAGGCAGCGGTG  *pCXU-HA-MaAS1^Ser61E, Thr92E, Ser120E^-R*: TATTGCCAAATGTTTGAACGATCTCACCGCCCATGCGTCTC  *pCXU-HA-MaAS1^Ser61A, Thr92A, Ser120A^-F*: CATACGATGTTCCAGATTACGCTATGAAGGATAGGCAGCGGTG  *pCXU-HA-MaAS1^Ser61A, Thr92A, Ser120A^-R*: TATTGCCAAATGTTTGAACGATCTCACCGCCCATGCGTCTC  *pCXU-HA-MaRNF4-F*: CATACGATGTTCCAGATTACGCTATGAGCACCCTTGGACCATCAA  *pCXU-HA-MaRNF4-R*: TATTGCCAAATGTTTGAACGATCCTACTTGGATGATGGAAGATACACAC  *pCXU-HA-MaMPK3-2-F*: CATACGATGTTCCAGATTACGCTATGGACGGAGCTCCGGCGAAC  *pCXU-HA-MaMPK3-2-R*: TATTGCCAAATGTTTGAACGATCTCAGCACTTGTAGTCTGGATTGAAC | | *Xcm* I  *Xcm* I  *Xcm* I  *Xcm* I  *Xcm* I  *Xcm* I  *Xcm* I  *Xcm* I  *Xcm* I  *Xcm* I |
| BiFC assay | *MaAS1-YC/NE-F*:  GGCGCGCCACTAGTGGATCCATGAAGGATAGGCAGCGGTG  *MaAS1-YC/NE-R*:  GCGGTACCCTCGAGGTCGACCCGCCCATGCGTCTC  *MaRNF4-YC/NE-F*: GGCGCGCCACTAGTGGATCCATGAGCACCCTTGGACCATCAA  *MaRNF4-YC/NE-R*: GCGGTACCCTCGAGGTCGACCTTGGATGATGGAAGATACACAC  *MaMPK3-2-YC/NE-F*: GGCGCGCCACTAGTGGATCCATGGACGGAGCTCCGGCGAAC  *MaMPK3-2-YC/NE-R*: GCGGTACCCTCGAGGTCGACGCACTTGTAGTCTGGATTGAAC  *MaMPK9-1-YC/NE-F*: GGCGCGCCACTAGTGGATCCATGGGGGGAGGAGCTCTCGTG  *MaMPK9-1-YC/NE-R*: GCGGTACCCTCGAGGTCGACAACATACAGTTCCTCCACCTTAT  *MaMPK11-2A-YC/NE-F*: GGCGCGCCACTAGTGGATCCATGATGGAGTCCGGGTCCGCC  *MaMPK11-2A-YC/NE-R*: GCGGTACCCTCGAGGTCGACGTGCACTACTGGATCTGGGTT  *MaMPK11-3-YC/NE-F*: GGCGCGCCACTAGTGGATCCATGGATTGCGGGTCGGCAG  *MaMPK11-3-YC/NE-R*: GCGGTACCCTCGAGGTCGACGTATACTGGATCTGGGTTGAAC  *MaMPK20-4-YC/NE-F*: GGCGCGCCACTAGTGGATCCATGCCGCAAGACCAGCGAAAGAA  *MaMPK20-4-YC/NE-R*: GCGGTACCCTCGAGGTCGACAGACATCCTTGACACGCTACG  *MaMPK9-4-YC/NE-F*: GGCGCGCCACTAGTGGATCCATGCTTCCTCCATCTCGAAGGGA  *MaMPK9-4-YC/NE-R*: GCGGTACCCTCGAGGTCGACAGAATATAACTGAGCCAACTTGTG | | *Bam*H I  *Sal* I  *Bam*H I  *Sal* I  *Bam*H I  *Sal* I  *Bam*H I  *Sal* I  *Bam*H I  *Sal* I  *Bam*H I  *Sal* I  *Bam*H I  *Sal* I  *Bam*H I  *Sal* I |
| Genetic transformation | *pBI121-MaAS1-F*:  GAGAACACGGGGGACTCTAGAATGAAGGATAGGCAGCGGTG  *pBI121-MaAS1-R*:  ATCGGTACCCTCGAGGGATCCCCGCCCATGCGTCTC  *pBI121-MaAS1^Ser61E, Thr92E, Ser120E^-F*: GAGAACACGGGGGACTCTAGAATGAAGGATAGGCAGCGGTG  *pBI121-MaAS1^Ser61E, Thr92E, Ser120E^-R*: ATCGGTACCCTCGAGGGATCCCCGCCCATGCGTCTC  *pBI121-MaAS1^Ser61A, Thr92A, Ser120A^-F*: GAGAACACGGGGGACTCTAGAATGAAGGATAGGCAGCGGTG  *pBI121-MaAS1^Ser61A, Thr92A, Ser120A^-R*: ATCGGTACCCTCGAGGGATCCCCGCCCATGCGTCTC  *pBI121-MaRNF4-F*: GAGAACACGGGGGACTCTAGAATGAGCACCCTTGGACCATCAA  *pBI121-MaRNF4-R*: ATCGGTACCCTCGAGGGATCCCTTGGATGATGGAAGATACACAC  *pBI121-MaMPK3-2-F*: GAGAACACGGGGGACTCTAGAATGGACGGAGCTCCGGCGAAC  *pBI121-MaMPK3-2-R*: ATCGGTACCCTCGAGGGATCCGCACTTGTAGTCTGGATTGAAC | | *Xbal* I  *Kpn* I  *Xbal* I  *Kpn* I  *Xbal* I  *Kpn* I  *Xbal* I  *Kpn* I  *Xbal* I  *Kpn* I |
| *Vivo* ubiquitination | *MaAS1-pEAQ-GFP-F*: CAAATTCGCGACCGGTATGAAGGATAGGCAGCGGTG  *MaAS1-pEAQ-GFP-R*: TGCTAGTCATACCGGTCCGCCCATGCGTCTC  *MaRNF4-pEAQ-His-F*: TTCTGCCCAAATTCGCGACCGGTATGAGCACCCTTGGACCATCAA  *MaRNF4-pEAQ-His-R*: TGATGGTGATGGTGATGCCCGGGCTTGGATGATGGAAGATACACAC | | *Age* I  *Age* I  *Age* I  *Xma* l |
